# A Galaxy-based training resource for single-cell RNA-sequencing quality control and analyses

**DOI:** 10.1093/gigascience/giz144

**Published:** 2019-12-11

**Authors:** Graham J Etherington, Nicola Soranzo, Suhaib Mohammed, Wilfried Haerty, Robert P Davey, Federica Di Palma

**Affiliations:** 1 Earlham Institute, Norwich Research Park, Norwich NR4 7UZ, UK; 2 European Bioinformatics Institute, Wellcome Genome Campus, Hinxton, Cambridgeshire CB10 1SD, UK

**Keywords:** scRNA-seq, single cell, scater, Galaxy, training

## Abstract

**Background:**

It is not a trivial step to move from single-cell RNA-sequencing (scRNA-seq) data production to data analysis. There is a lack of intuitive training materials and easy-to-use analysis tools, and researchers can find it difficult to master the basics of scRNA-seq quality control and the later analysis.

**Results:**

We have developed a range of practical scripts, together with their corresponding Galaxy wrappers, that make scRNA-seq training and quality control accessible to researchers previously daunted by the prospect of scRNA-seq analysis. We implement a “visualize-filter-visualize” paradigm through simple command line tools that use the Loom format to exchange data between the tools. The point-and-click nature of Galaxy makes it easy to assess, visualize, and filter scRNA-seq data from short-read sequencing data.

**Conclusion:**

We have developed a suite of scRNA-seq tools that can be used for both training and more in-depth analyses.

## Background

The advent of RNA sequencing (RNA-seq) has enabled a host of important discoveries in many biological areas such as gene expression, alternative splicing, comparative genomics, and gene annotation. Bulk RNA-seq, where a population of cells is used in every sample, usually provides copious amounts of RNA but only measures the average expression level across that population. If different cell populations are included in a single sample, then information may be missed owing to the transcription profile of one population dominating another. A decade ago, the development of single-cell RNA-seq (scRNA-seq) made it possible to sequence the transcriptome of individual cells [1]. This innovation opened the door to the identification of novel cell types, uncovering regulatory pathways between genes, tracing the trajectories of distinct cell lineages, and pseudo-time reconstruction [2].

Typically, reads generated from cells in scRNA-seq experiments are mapped to a reference genome and then an expression matrix is calculated from the number of reads that are allocated to each gene or transcript. Owing to both the large amount of sequencing data that scRNA-seq may produce and the high computational resources required by many of the tools, specialized infrastructure such as high-performance computing (HPC) is often required to analyse such experiments. There are a number of tools available that use HPC platforms to perform these processes (or variations of them) that are widely used by bioinformaticians [3–5].

Along with the advantages of scRNA-seq come a number of technical challenges. scRNA-seq data are inherently noisy. Disruption or damage to the cell can result in the escape or degradation of nuclear DNA, leaving predominantly cytoplasmic DNA in the cell. Furthermore, inefficient RNA capture combined with amplification bias may distort gene expression profiles. This often results in “dropouts,” where genes are found to be at least moderately expressed in a few cells, but absent from most.

A large array of tools are now available to address quality control (QC) and expression analysis in scRNA-seq data (e.g., [6–12]). The scater package (part of the Bioconductor collection) provides the capacity to QC scRNA-seq data, providing methods for visualization, filtering, and expression analyses [13, 14]. To facilitate the compatibility with further downstream analysis tools, datasets produced by scater can be exported into the Loom format using the LoomExperiment package. Loom is an efficient file format for handling large ‘omics datasets [15]. It is ideal for handling scRNA-seq data, along with all associated metadata. Because Loom provides efficient access to arbitrary rows and columns, it scales well with the increasing size of high-throughput genomics data and is supported by nearly all programming languages.

Like many of the other tools, scater requires at least some experience of the R programming language [16]. There is a lack of training resources for wet-lab scientists who want to carry out computational analyses of next-generation sequencing data. Despite online resources for programming being quite plentiful along with a plethora of online help forums, resources focused on both training and analyses of biological data are few [17].

Galaxy is an open source scientific workflow, data integration, and data analysis platform that aims to make life science research accessible to research scientists who do not have computer programming or systems administration experience [18]. Galaxy is available through >150 public Galaxy servers and can be easily integrated into existing HPC and cloud resources.

More than 30 scientific groups involved in Galaxy-related training contribute to the Galaxy Training Network (GTN) [19]. The GTN provides online training materials, as well as coordinating Galaxy training events worldwide [20].

## Results

### scater wrappers and workflows

Here we describe our scater command line wrappers, along with their integration into Galaxy and associated training material, for use as both an introduction to scRNA-seq data analyses and for more focused data QC.

**Figure 1:**
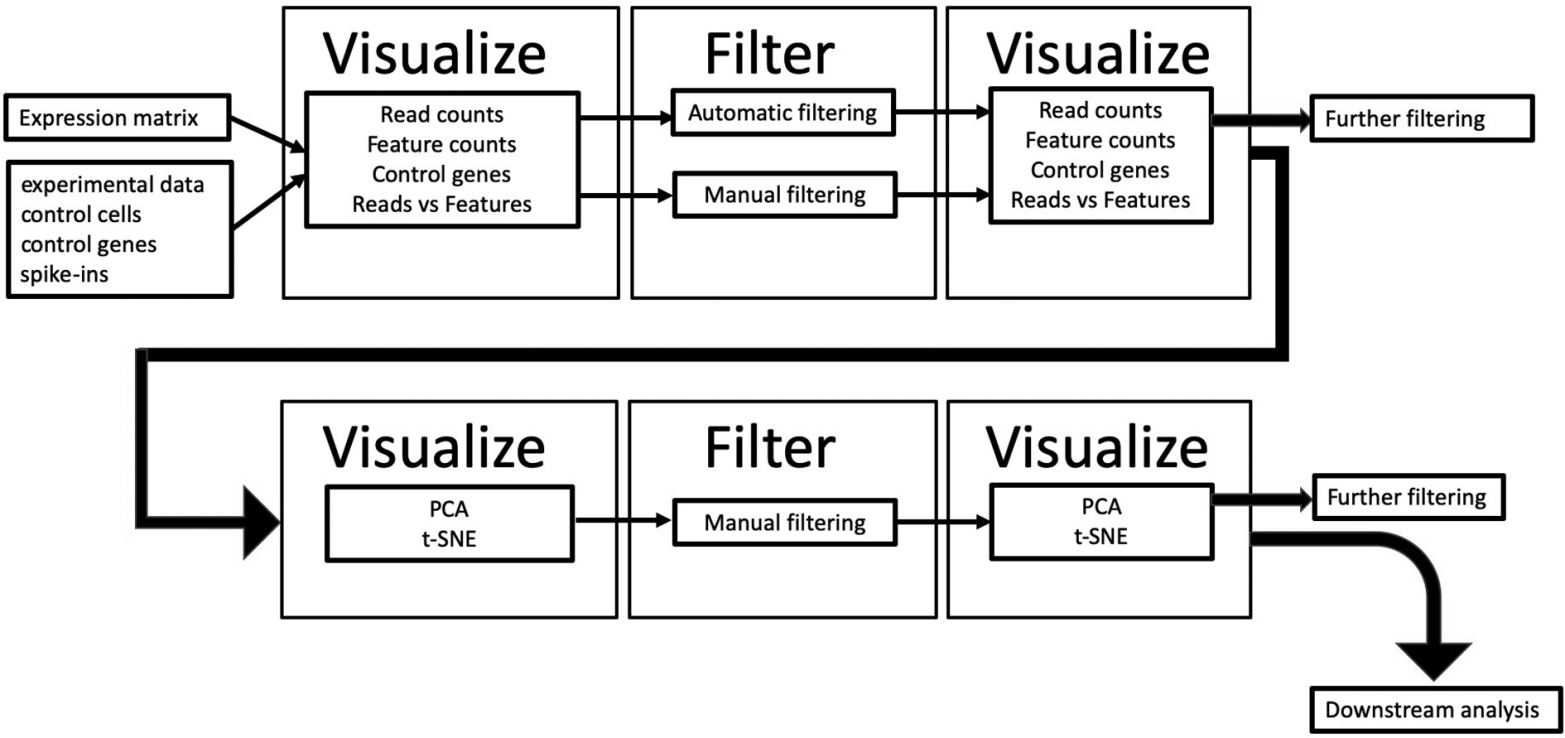
The visualize-filter-visualize paradigm allows users to go from raw data to high-quality analysis-ready data. First, read counts, feature counts (e.g., transcripts, genes), and control genes are visualized to inform filtering parameters. The dataset is then filtered and then re-visualized to examine the effect the previous filtering step had on the data. Further steps of filtering can be carried out until only high-quality cells remain. Next, batch effects are examined using the same paradigm but with principal component analysis (PCA) or t-stochastic neighbour embedding (t-SNE) plots. By the end of the workflow, only high-quality data ready for downstream analysis should remain.

Using scater v1.10.1 we defined the most common and intuitive tasks that researchers new to the field of scRNA-seq might carry out in their analyses and created generic tools to accomplish these tasks [13]. Typically, one would read in an expression matrix, calculate metrics on the data, visualize the data, and then filter out low-quality cells or unexpressed genes. Then, the same metrics would be recalculated and visualized to assess the impact of the previous filtering steps. This visualize-filter-visualize iteration can be continued until a user is satisfied that they have retained only high-quality data. The next step would be to look for confounding factors in the data, such as batch effects, by clustering the data and plotting it in relation to experimental metadata, or any other non-biological variables that might have an effect on the final data. Such factors could include which plate each cell was generated on, the sequencing run, extraction date, laboratory technician, batch, replicate, etc. (Fig. 1).

Using methods from the scater suite, along with other bespoke analysis and plotting methods, we have interpreted these tasks into a number of easy-to-use command line scripts, which require only the most basic familiarity with the command line. Furthermore, these scripts may be integrated into the Galaxy platform by using the Galaxy wrappers developed alongside the scripts. We also provide the inbuilt scater data as a range of input files for users to input and test the methods outlined below.

The basic workflow is as follows:

Step 1. The user inputs the data (a sample × gene read count matrix), along with other metadata such as the experimental annotation and any control genes (often ERCC spike-ins or a list of mitochondrial genes). This is then loaded into scater and a number of QC metrics are automatically calculated on the data. The output from this is a Loom file [15], an HDF5-based format that is designed to efficiently store large 'omics datasets. These Loom files are then used as the input for all subsequent steps in the workflow (Supplementary Fig. S1).

**Figure 2:**
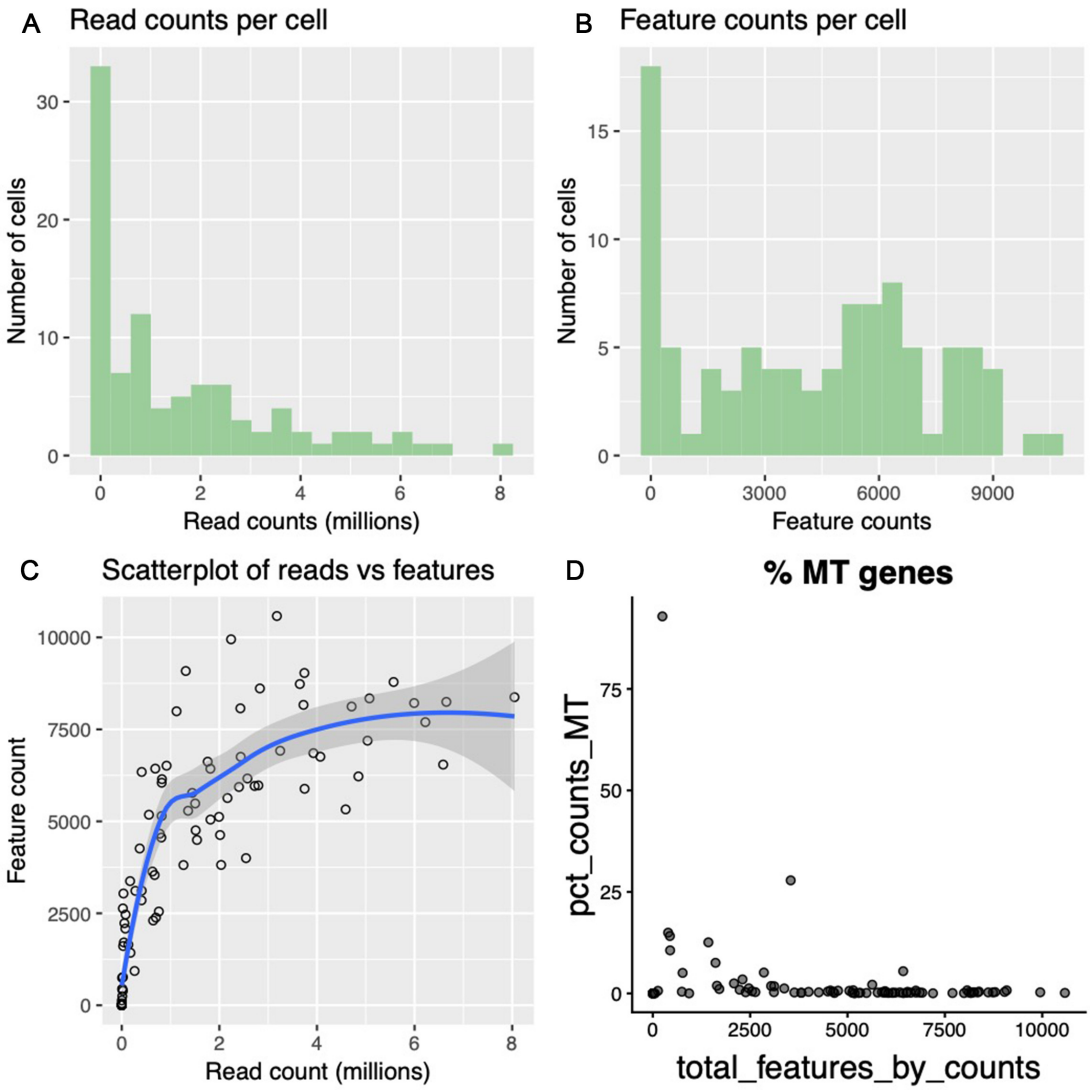
The plotting tools provide the ability to visualize the quality of each cell in the form of histograms and scatterplots. Raw data, before filtering, are plotted in this figure. Typically, raw data will show a high number of cells with both low read counts (read counts per cell, Fig. 2A) and low feature counts (feature counts per cell, Fig. 2B). Low-quality cells often have both low read and feature counts, depicted by the cluster of cells at the base of the x and y axes in “scatterplot of reads vs features” (Fig. 2C). Last, the “% MT genes” plot shows the proportion of reads mapping to mitochondrial genes (Fig. 2D). We can see that ≥1 cell has >75% of its reads mapped to mitochondrial genes, suggesting that the cell was degraded.

Step 2. The data are visualized using a range of plots to show information about each cell (Fig. 2). The distribution of reads in each cell, the number of genes expressed in each cell, and a scatterplot of the number of reads vs the number of expressed genes are all plotted. Finally, the percentage of mitochondrial genes in proportion to the total number of genes expressed is also plotted. These visualizations provide insight into poor-quality cells that have either low read counts, low gene counts, or high mitochondrial gene expression (Supplementary Fig. S2).

Step 3. The data can now be filtered in 2 different ways. The user can decide to use information from the visualizations to, for example, remove cells that have low read count, or the user can use a principal component analysis (PCA) filtering method where cells calculated to be outliers are removed automatically. The output from this step is a new Loom file with the low-quality cells filtered out.

**Figure 3:**
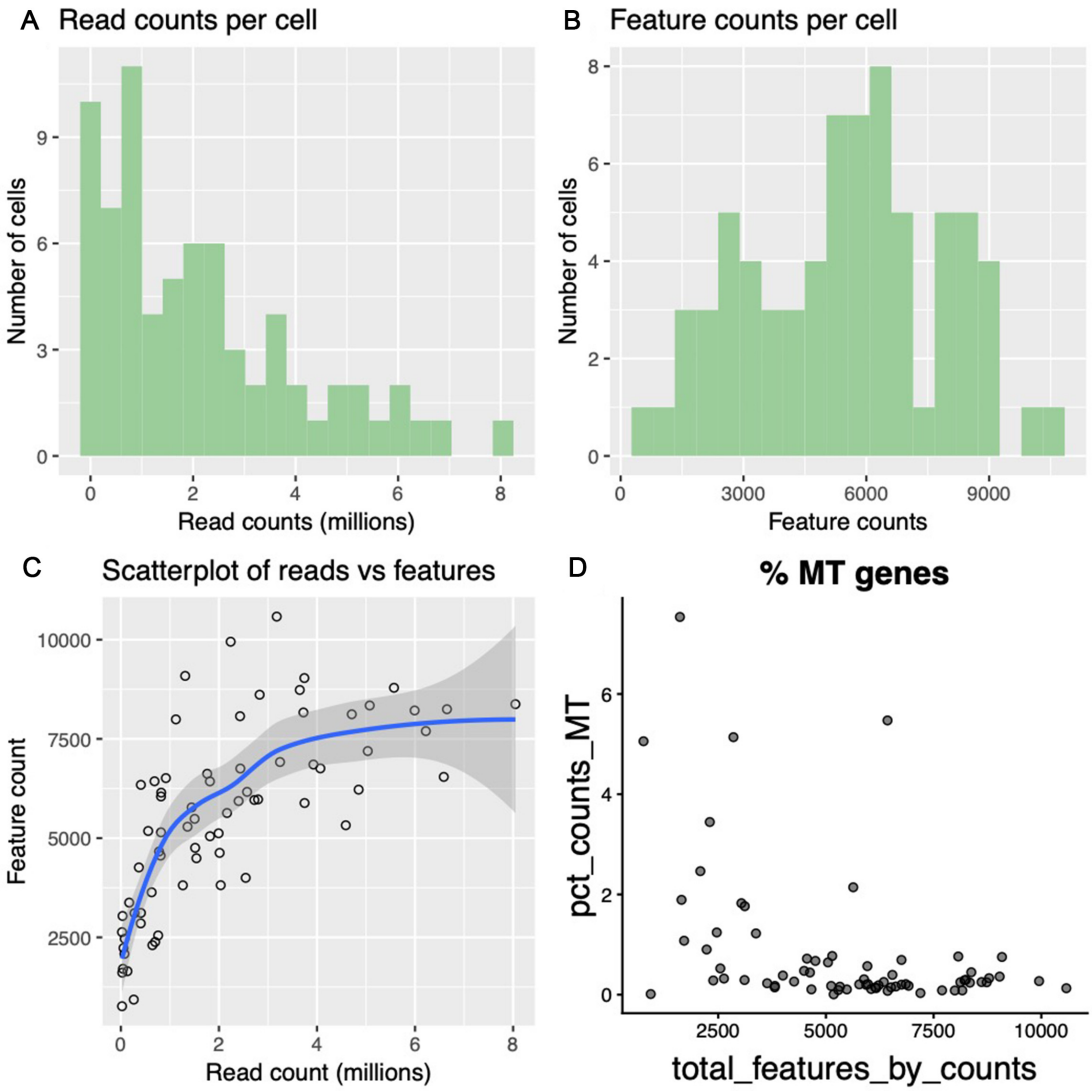
Plotting of data after QC. The data shown in Fig. 2 have been filtered by removing cells that have <10,000 mapped reads and cells that have >8% of their expressed features from mitochondrial DNA. Most of the low-quality cells have been removed, leaving cells that have a high number of reads (Fig. 3A) and features (genes in this case) (Fig. 3B) and a low percentage of mitochondrial genes (Fig. 3D). Note that the tight cluster of cells at the base of the x and y axes in “scatterplot of reads vs features” (Fig. 3C) has now also disappeared. From the original 95 cells in the pre-filtered dataset, we have 71 high-quality cells remaining.

Step 4. The filtered data can now be visualized (as in step 2 above) to assess the filtering process carried out in step 3. These 2 steps can be carried out iteratively, steadily increasing the parameters until the user is satisfied that they have only the highest-quality cells remaining (Fig. 3).

Step 5. Once the user has a high-quality dataset, they can investigate any confounding factors in the data, such as batch effect. Any metric in the experiment annotation file may be plotted and variation in metrics or categories may be displayed by setting the size, colour, or shape of the plotted points. As in the previous steps, this can be carried out iteratively until only high-quality data ready for downstream analysis remain.

These steps are further explained via a tutorial with training data at the Galaxy Training Materials website [20]. Each of the above steps is covered in greater detail, along with additional information and guidance for interpreting the output from each tool.

## Methods

We present a 5-step workflow in our results above for which we use “ready-made” scater methods, along with our own bespoke methods.

### Data input

When data are read in, a number of sanity checks take place to confirm that the minimum required information has been loaded and then further decisions are made depending on what additional information is loaded (e.g., have ERCC spike-ins been included in the data?). These raw data are then used to calculate expression metrics using scater’s “calculateQCMetrics” method. The output is then saved as a Loom file.

### Plotting tools

There are a number of plotting tools provided to the user. One such tool uses ggplot2 (ggplot2, RRID:SCR_014601) to lay out a panel of 4 plots containing a scatterplot of mitochondrial gene expression, 2 histograms depicting read counts and feature counts (e.g., genes, transcripts), and finally a scatterplot of read counts vs feature counts, which plots a smoothed regression line overlaid by the 95% confidence interval to provide the uncertainty about the regression line [21]. An additional tool is provided that uses scater’s (scater, RRID:SCR_015954) inbuilt “plotExprsFreqVsMean” method, which plots gene expression frequency against mean expression level, in order to examine the effects of technical dropout in the data. Finally, we provide methods to examine batch effect and other confounding factors in our QC-adhering data. A confounding factor might be any parameter introduced during library preparation or sequencing, such as the cells being from different sequencing runs, being handled by different technicians, or being processed on different days. Using scater’s inbuilt methods, the high-quality data are first normalized. Next, a PCA is applied to the normalized data, which is then plotted. Points on the plot represent data that explain the maximal amount of variance, which can be annotated in relation to any column heading in the experiment annotation file. Categorical data may be given a unique shape or colour, whilst points for continuous data may be given scaled sizes. For example, points on the PCA plot may be coloured by sample, shaped by batch, and sized by total features. Because batch effects can be hidden in higher-order components, we also provide a method to generate t-stochastic neighbour embedding (t-SNE) plots. Using normalized data, the t-SNE for the cells is first calculated and then plotted, again with the ability to shape, size, and colour points according to experimental parameters.

### Filtering tool

We provide 2 alternative methods for filtering. In the first one the user manually selects cut-off parameters (usually informed by the plotting tools above), above or below which cells are removed if they do not reach the threshold. The metrics that can be filtered for are as follows: the number of expressed genes, library size (calculated from the number of mapped reads), and percentage of reads mapped to mitochondrial genes. Cells failing these thresholds will be removed. The second method automatically removes cells that are categorized as outliers from PCA. This method works by identifying low-quality cells that have markedly different QC metrics from other cells. Both of the filtering methods are designed to be iterative, and the user has the option to re-run filtering from raw data or refine filtering from a previous filtering step (Fig. 1).

### Future work

Although we do not include an exhaustive suite of tools, we aim to include ones that are being used widely in scRNA-seq QC. Future tools would most likely include improved outlier detection methods along with methods to account for cell cycle heterogeneity.

## Conclusion

A large number of tools are available to analyse scRNA-seq data, but many of them require quite advanced computational skills and resources to run. These requirements make it difficult to first learn the basics of scRNA-seq analyses and to then have the power to perform more complicated downstream analyses on typically large datasets (hundreds or even thousands of samples). We have developed tools and training materials that make it easy to learn and run typical short-read scRNA-seq QC steps and analyses. These tools can either be used on the command line in an intuitive, iterative manner, or be integrated into the Galaxy platform, which will allow further downstream analyses with other tools.

## Availability of Supporting Source Code and Requirements

- Project name: Wrappers for scater
- Project home page: https://github.com/galaxyproject/tools-iuc/tree/master/tools/scater
- Operating systems: Platform independent
- Programming language: R
- Other requirements: scater
- License: MIT
- RRID:SCR_017394

## Availability of Supporting Data and Materials

All of our code and wrappers, complete with installation instructions, tool help, and an example workflow, are available under the MIT open source license at https://github.com/galaxyproject/tools-iuc/tree/master/tools/scater and on the Galaxy ToolShed at https://toolshed.g2.bx.psu.edu/view/iuc/suite_scater/. Snapshots of the code are also available from the *GigaScience* GigaDB repository [22]. The tools can also be freely used at the UseGalaxy.eu public server (https://usegalaxy.eu). We have added a tutorial that covers a typical analysis workflow in Galaxy, which can be found at https://training.galaxyproject.org/training-material/topics/transcriptomics/tutorials/scrna-scater-qc/tutorial.html.

This resource has been submitted to SciCrunch.org under RRID:SCR_017394 and is tracked in bio.tools under software name “Galaxy scater.”

## Additional Files

**Supplementary Figure S1**. The Galaxy interface for the scater tools, shown in the left-hand column. Data are shown in green in the history panel to the right of the interface. Here, an expression matrix, cell annotation, and mitochondrial control genes have been used to create QC-ready data by using the “Calculate QC metrics” tool. The “plot library QC” tool has now been selected, and the user merely selects the input file to run the tool.

**Supplementary Figure S2**. The output of the “plot library QC” tool can be displayed within Galaxy. The plot is used to inform parameters for the subsequent filtering steps.

giz144_GIGA-D-19-00275_Original_Submission

giz144_GIGA-D-19-00275_Revision_1

giz144_Response_to_Reviewer_Comments_Original_Submission

giz144_Reviewer_1_Report_Original_Submission Mehmet Tekman -- 8/6/2019 Reviewed

giz144_Reviewer_2_Report_Original_Submission Wendi Bacon -- 8/15/2019 Reviewed

giz144_Reviewer_3_Report_Original_Submission Rory Kirchner -- 8/16/2019 Reviewed

giz144_Supplemental_Figures

## Abbreviations

GTN: Galaxy Training Network; HPC: high-performance computing; PCA: principal component analysis; QC: quality control; RNA-seq: RNA sequencing; scRNA-seq: single-cell RNA sequencing; t-SNE: t-stochastic neighbour embedding.

## Competing Interests

The authors declare that they have no competing interests.

## Funding

This work was strategically funded by the BBSRC Core Strategic Programme Grants BBS/E/T/000PR9817, BBS/E/T/000PR9818, and BBS/E/T/000PR9819 and Core Capability Grant BBS/E/T/000PR9816 at the Earlham Institute.

## References

[1] Tang F, Barbacioru C, Wang Y, et al. mRNA-Seq whole-transcriptome analysis of a single cell. Nat Methods. 2009;6(5):377–82., doi:10.1038/nmeth.1315. 19349980

[2] Wagner A, Regev A, Yosef N Revealing the vectors of cellular identity with single-cell genomics. Nat Biotechnol. 2016;34(11):1145–60., doi:10.1038/nbt.3711. 27824854 PMC5465644

[3] Bray NL, Pimentel H, Melsted P, et al. Near-optimal probabilistic RNA-seq quantification. Nat Biotechnol. 2016;34(5):525–7., doi:10.1038/nbt.3519. 27043002

[4] Dobin A, Davis CA, Schlesinger F, et al. STAR: ultrafast universal RNA-seq aligner. Bioinformatics. 2013;29(1):15–21., doi:10.1093/bioinformatics/bts635. 23104886 PMC3530905

[5] Patro R, Duggal G, Love MI, et al. Salmon provides fast and bias-aware quantification of transcript expression. Nat Methods. 2017;14(4):417–9., doi:10.1038/nmeth.4197. 28263959 PMC5600148

[6] Andrews TS, Hemberg M M3Drop: dropout-based feature selection for scRNASeq. Bioinformatics. 2019;35:2865–7., doi:10.1093/bioinformatics/bty1044. 30590489 PMC6691329

[7] Butler A, Hoffman P, Smibert P, et al. Integrating single-cell transcriptomic data across different conditions, technologies, and species. Nat Biotechnol. 2018;36(5):411–20., doi:10.1038/nbt.4096. 29608179 PMC6700744

[8] Ji Z, Ji H TSCAN: Pseudo-time reconstruction and evaluation in single-cell RNA-seq analysis. Nucleic Acids Res. 2016;44(13):e117, doi:10.1093/nar/gkw430. 27179027 PMC4994863

[9] Kiselev VY, Kirschner K, Schaub MT, et al. SC3: consensus clustering of single-cell RNA-seq data. Nat Methods. 2017;14(5):483–6., doi:10.1038/nmeth.4236. 28346451 PMC5410170

[10] Qiu X, Mao Q, Tang Y, et al. Reversed graph embedding resolves complex single-cell trajectories. Nat Methods. 2017;14(10):979–82., doi:10.1038/nmeth.4402. 28825705 PMC5764547

[11] Smith T, Heger A, Sudbery I UMI-tools: modeling sequencing errors in Unique Molecular Identifiers to improve quantification accuracy. Genome Res. 2017;27(3):491–9., doi:10.1101/gr.209601.116. 28100584 PMC5340976

[12] Wolf FA, Angerer P, Theis FJ SCANPY: large-scale single-cell gene expression data analysis. Genome Biol. 2018;19(1):15, doi:10.1186/s13059-017-1382-0. 29409532 PMC5802054

[13] McCarthy DJ, Campbell KR, Lun AT, et al. Scater: pre-processing, quality control, normalization and visualization of single-cell RNA-seq data in R. Bioinformatics. 2017;33(8):1179–86., doi:10.1093/bioinformatics/btw777. 28088763 PMC5408845

[14] Gentleman RC, Carey VJ, Bates DM, et al. Bioconductor: open software development for computational biology and bioinformatics. Genome Biol. 2004;5(10):R80, doi:10.1186/gb-2004-5-10-r80. 15461798 PMC545600

[15] Loompy. http://loompy.org/. Accessed 1 July 2019.

[16] R Core Team. R: A Language and Environment for Statistical Computing 2018 https://www.r-project.org/. Accessed 1 July 2019.

[17] Kwok R Computing: out of the hood. Nature. 2013;504(7479):319–21. 24350353 10.1038/nj7479-319a

[18] Afgan E, Baker D, Batut B, et al. The Galaxy platform for accessible, reproducible and collaborative biomedical analyses: 2018 update. Nucleic Acids Res. 2018;46(W1):W537–44., doi:10.1093/nar/gky379. 29790989 PMC6030816

[19] Galaxy Training Network. https://galaxyproject.org/teach/gtn/. Accessed 1 July 2019.

[20] Batut B, Hiltemann S, Bagnacani A, et al. Community-driven data analysis training for biology. Cell Syst. 2018;6(6):752–758 e1., doi:10.1016/j.cels.2018.05.012. 29953864 PMC6296361

[21] Wickham H ggplot2: Elegant Graphics for Data Analysis. New York, NY: Springer; 2016.

[22] Etherington GJ, Soranzo N, Mohammed S, et al. Supporting data for “A Galaxy-based training resource for single-cell RNA-sequencing quality control and analyses.”. GigaScience Database. 2019 10.5524/100663. Accessed 1 July 2019. PMC6905351 31825480

