# Supplementary material for: A Galaxy-based training resource for single-cell RNA-sequencing quality control and analyses
Source: Gigascience. 2019 Dec 11;8(12):giz144. doi: 10.1093/gigascience/giz144 (PMC6905351; doi:10.1093/gigascience/giz144)
Supplement: giz144_GIGA-D-19-00275_Revision_1 [file giz144_giga-d-19-00275_revision_1.pdf]

GigaScience

A Galaxy-based training resource for single-cell RNA-seq quality control and analyses

--Manuscript Draft--

**Manuscript Number:**	GIGA-D-19-00275R1
**Full Title:**	A Galaxy-based training resource for single-cell RNA-seq quality control and analyses
**Article Type:**	Technical Note
**Funding Information:**	Biotechnology and Biological Sciences Research Council (BBS/E/T/000PR9816) — Not applicable<br>Biotechnology and Biological Sciences Research Council (BBS/E/T/000PR9818) — Prof. Federica Di Palma<br>Biotechnology and Biological Sciences Research Council (BBS/E/T/000PR9819) — Prof. Federica Di Palma<br>Biotechnology and Biological Sciences Research Council (BBS/E/T/000PR9817) — Prof. Federica Di Palma
**Abstract:**	Background<br>It is not a trivial step to move from single-cell RNA-seq (scRNA-seq) data production to data analysis. There is a lack of intuitive training materials and easy-to-use analysis tools, and researchers can find it difficult to master the basics of scRNA-seq quality control and the later analysis.<br><br>Results<br>We have developed a range of practical scripts, together with their corresponding Galaxy wrappers, that make scRNA-seq training and quality-control accessible to researchers previously daunted by the prospect of scRNA-seq analysis. We implement a ‘visualise-filter-visualise’ paradigm through simple command-line tools that use the Loom format to exchange data between the tools. The point-and-click nature of Galaxy makes it easy to assess, visualise, and filter scRNA-seq data from short-read sequencing data.<br><br>Conclusion<br>We have developed a suite of scRNA-seq tools that can be used for both training and more in-depth analyses.
**Corresponding Author:**	Graham John Etherington, BSc, PhD<br>Earlham Institute<br>Norwich, Norfolk UNITED KINGDOM
**Corresponding Author Secondary Information:**	
**Corresponding Author's Institution:**	Earlham Institute
**Corresponding Author's Secondary Institution:**	
**First Author:**	Graham John Etherington, BSc, PhD
**First Author Secondary Information:**	
**Order of Authors:**	Graham John Etherington, BSc, PhD<br>Nicola Soranzo<br>Suhaib Mohammed<br>Wilfried Haerty<br>Robert P Davey<br>Federica Di Palma

Powered by Editorial Manager® and ProduXion Manager® from Aries Systems Corporation

**Order of Authors Secondary Information:**	
**Response to Reviewers:**	Please see personal cover file, 'ResponseToReviewers'.
**Additional Information:**	
**Question**	**Response**
Are you submitting this manuscript to a special series or article collection?	No
**Experimental design and statistics**<br><br>Full details of the experimental design and statistical methods used should be given in the Methods section, as detailed in our Minimum Standards Reporting Checklist. Information essential to interpreting the data presented should be made available in the figure legends.<br><br>Have you included all the information requested in your manuscript?	Yes
**Resources**<br><br>A description of all resources used, including antibodies, cell lines, animals and software tools, with enough information to allow them to be uniquely identified, should be included in the Methods section. Authors are strongly encouraged to cite Research Resource Identifiers (RRIDs) for antibodies, model organisms and tools, where possible.<br><br>Have you included the information requested as detailed in our Minimum Standards Reporting Checklist?	Yes
**Availability of data and materials**<br><br>All datasets and code on which the conclusions of the paper rely must be either included in your submission or deposited in publicly available repositories (where available and ethically appropriate), referencing such data using a unique identifier in the references and in	Yes

Powered by Editorial Manager® and ProduXion Manager® from Aries Systems Corporation

the "Availability of Data and Materials" section of your manuscript.<br><br>Have you have met the above requirement as detailed in our Minimum Standards Reporting Checklist?	

Manuscript

Click here to view linked References

Click here to access/download;Manuscript;Manuscript_resub_Galaxyscater_

A Galaxy-based training resource for single-cell RNA-seq quality control and analyses.

Graham J Etherington[1], Nicola Soranzo[1], Suhaib Mohammed[2], Wilfried Haerty[1], Robert P Davey[1], Federica Di Palma[1].

1. Earlham Institute, Norwich Research Park, Norwich NR4 7UZ, United Kingdom

2. The European Bioinformatics Institute, Wellcome Genome Campus, Hinxton, Cambridgeshire, CB10 1SD, United Kingdom

Corresponding author: graham.etherington@earlham.ac.uk

ORCIDs:

Graham Etherington: 0000-0002-5003-1425; Nicola Soranzo: 0000-0003-3627-5340; Suhaib Mohammed: 0000-0003-1772-8690; Wilfried Haerty: 0000-0003-0111-191X; Robert Davey: 0000-0002-5589-7754; Federica Di Palma: 0000-0002-4394-0102

Keywords: scRNA-seq, single cell, *scater*, Galaxy, training.

Abstract

Background

It is not a trivial step to move from single-cell RNA-seq (scRNA-seq) data production to data analysis. There is a lack of intuitive training materials and easy-to-use analysis tools, and researchers can find it difficult to master the basics of scRNA-seq quality control and the later analysis.

Results

We have developed a range of practical scripts, together with their corresponding Galaxy wrappers, that make scRNA-seq training and quality-control accessible to researchers previously daunted by the prospect of scRNA-seq analysis. We implement a ‘visualise-filter-

visualise' paradigm through simple command-line tools that use the Loom format to exchange data between the tools. The point-and-click nature of Galaxy makes it easy to assess, visualise, and filter scRNA-seq data from short-read sequencing data.

Conclusion

We have developed a suite of scRNA-seq tools that can be used for both training and more in-depth analyses.

Background

The advent of RNA-seq has enabled a host of important discoveries in many biological areas such as gene expression, alternative splicing, comparative genomics, and gene annotation. Bulk RNA-seq, where a population of cells is used in every sample, usually provides copious amounts of RNA, but only measures the average expression level across that population. If different cell populations are included in a single sample, then information may be missed due to the transcription profile of one population dominating another. A decade ago, the development of single-cell RNA-seq (scRNA-seq) made it possible to sequence the transcriptome of individual cells [1]. This innovation opened the door to the identification of novel cell types, uncovering regulatory pathways between genes, tracing the trajectories of distinct cell lineages, and pseudo-time reconstruction [2].

Typically, reads generated from cells in scRNA-seq experiments are mapped to a reference genome and then an expression matrix is calculated from the number of reads that are allocated to each gene or transcript. Due to both the large amount of sequencing data that scRNA-seq may produce and the high computational resources required by many of the tools, specialised infrastructure such as High-Performance Computing (HPC) is often required to

analyse such experiments. There are a number of tools available that utilise HPC platforms to perform these processes (or variations of them) that are widely used by bioinformaticians [3-5].

Along with the advantages of scRNA-seq come a number of technical challenges. scRNA-seq data are inherently noisy. Disruption or damage to the cell can result in the escape or degradation of nuclear DNA leaving predominantly cytoplasmic DNA in the cell. Further, inefficient RNA capture combined with amplification bias may distort gene expression profiles. This often results in ‘dropouts’, where genes are found to be at least moderately expressed in a few cells, but absent from most.

A large array of tools are now available to address quality control (QC) and expression analysis in scRNA-seq data (e.g. [6-12]). The *scater* package (part of the Bioconductor collection) provides the capacity to QC scRNA-seq data, providing methods for visualisation, filtering, and expression analyses [13, 14]. To facilitate the compatibility with further downstream analysis tools, data sets produced by *scater* can be exported into the Loom format using the LoomExperiment package. Loom is an efficient file format for handling large ‘omics datasets (http://loompy.org/). It is ideal for handling scRNA-seq data, along with all associated metadata. As Loom provides efficient access to arbitrary rows and columns, it scales well with the increasing size of high-throughput genomics data and is supported by nearly all programming languages.

Like many of the other tools, *scater* requires at least some experience of the R programming language [15]. There is a lack of training resources for wet-lab scientists who want to carry out computational analyses of NGS data. Despite online resources for programming being

quite plentiful along with a plethora of online help forums, resources focused on both training and analyses of biological data are few [16].

Galaxy is an open-source scientific workflow, data integration, and data analysis platform that aims to make life science research accessible to research scientists that do not have computer programming or systems administration experience [17]. Galaxy is available through over 150 public Galaxy servers and can be easily integrated into existing HPC and cloud resources.
Over 30 scientific groups involved in Galaxy-related training contribute to the Galaxy Training Network (GTN) (https://galaxyproject.org/teach/gtn/). The GTN provides online training materials as well as coordinating Galaxy training events worldwide [18].

Results

scater wrappers and workflows

Here we describe our *scater* command-line wrappers, along with their integration into Galaxy and associated training material, for use as both an introduction to scRNA-seq data analyses and for more focused data quality control.

Using *scater* v1.10.1 we defined the most common and intuitive tasks researchers new to the field of scRNA-seq might carry out in their analyses and created generic tools to accomplish these tasks [13]. Typically, one would read in an expression matrix, calculate metrics on the data, visualise the data and then filter out low quality cells or unexpressed genes. Then, the same metrics would be recalculated and visualised to assess the impact of the previous

filtering steps. This visualise-filter-visualise iteration can be continued until a user is happy they have retained only high-quality data. The next step would be to look for confounding factors in the data, such as batch effects, by clustering the data and plotting it in relation to experimental metadata, or any other non-biological variables that might have an effect on the final data. Such factors could include which plate each cell was generated on, the sequencing run, extraction date, lab technician, batch, replicate, etc. (Figure 1).

Figure 1. The visualise-filter-visualise paradigm allows users to go from raw data to high-quality analysis-ready data. First, read-counts, feature counts (transcripts, genes, etc.), and control genes are visualised to inform filtering parameters. The dataset is then filtered and then re-visualised to examine the effect the previous filtering step had on the data. Further steps of filtering can be carried out until only high-quality cells remain. Next, batch effects are examined using the same paradigm but with PCA or t-SNE plots. By the end of the workflow, only high-quality data ready for downstream analysis should remain.

Using methods from the *scater* suite, along with other bespoke analysis and plotting methods, we have interpreted these tasks into a number of easy-to-use command-line scripts, which requires only the most basic familiarity with the command line. Further, these scripts may be integrated into the Galaxy platform by using the Galaxy wrappers developed alongside the scripts. We also provide the inbuilt *scater* data as a range of input files for users to input and test the methods outlined below.

The basic workflow is as follows:

Step 1. The user inputs the data (a sample x gene read-count matrix), along with other meta-data such as the experimental annotation and any control genes (often ERCC spike-ins or a list of mitochondrial genes). This is then loaded into *scater* and a number of quality control metrics are automatically calculated on the data. The output from this is a Loom file (http://loompy.org/), an HDF5-based format which is designed to efficiently store large omics datasets. These Loom files are then used as the input for all subsequent steps in the workflow. (Supplementary data Figure S1)

Step 2. The data are visualised using a range of plots to show information about each cell (Figure 2). The distribution of reads in each cell, the number of genes expressed in each cell, and a scatterplot of the number of reads versus the number of expressed genes are all plotted. Finally, the percentage of mitochondrial genes in proportion to the total number of genes expressed is also plotted. These visualisations provide insight into poor quality cells that have either low read counts, low gene counts, or high mitochondrial gene expression (Supplementary data Figure S2).

Step 3. The data can now be filtered in two different ways. The user can decide to use information from the visualisations to, for example, remove cells that have low read count, or the user can use a PCA filtering method where cells calculated to be outliers are removed automatically. The output from this step is a new Loom file with the low-quality cells filtered-out.

Step 4. The filtered data can now be visualised (as in step 2 above) to assess the filtering process carried out in step 3. These two steps can be carried out iteratively, steadily increasing the parameters until the user is satisfied they have only the highest quality cells remaining (Figure 3).

Step 5. Once the user has a high-quality dataset, they can investigate any confounding factors in the data, such as batch effect. Any metric in the experiment annotation file may be plotted

and variation in metrics or categories may be displayed by setting the size, colour or shape of the plotted points. As in the previous steps, this can be carried out iteratively until only high-quality data ready for downstream analysis remain.

Figure 2. The plotting tools provide the ability to visualise the quality of each cell in the shape of histograms and scatterplots. Raw data, before filtering, are being plotted in this figure. Typically, raw data will show a high number of cells with both low read counts (Read counts per cell) and low feature counts (Feature counts per cell). Low quality cells often have both low read and feature counts, depicted by the cluster of cells at the base of the x and y axes in 'Scatterplot of reads vs features'. Lastly, the '% MT genes' plot shows the proportion of reads mapping to mitochondrial genes. We can see that at least one cell has more than 75% of its reads mapped to mitochondrial genes, suggesting the cell was degraded.

Figure 3. Plotting of data after quality control. The data shown in Figure 2 have been filtered by removing cells that have less than 10,000 mapped reads and cells that have more than 8% of their expressed features from mitochondrial DNA. Most of the low-quality cells have been removed, leaving cells that have a high number of reads and features (genes in this case) and a low percentage of mitochondrial genes. Note that the tight cluster of cells at the base of the x and y axes in "Scatterplot of reads vs features" has now also disappeared. From the original 95 cells in the pre-filtered dataset, we have 71 high-quality cells remaining.

These steps are further explained via a tutorial with training data at the Galaxy Training Materials website[18]. Each of the above steps are covered in greater detail, along with additional information and guidance for interpreting the output from each tool.

Methods

We present a 5-step workflow in our results above for which we use ‘ready-made’ *scater* methods, along with our own bespoke methods.

Data input

When data are read in, a number of sanity checks take place in order to confirm that the minimum required information has been loaded and then further decisions are made depending on what additional information is loaded (e.g. have ERCC spike-ins been included in the data). These raw data are then used to calculate expression metrics using *scater*’s ‘calculateQCMetrics’ method. The output is then saved as a Loom file.

Plotting tools

There are a number of plotting tools provided to the user. One such tool uses ggplot2 (ggplot2, RRID:SCR_014601) to layout a panel of four plots containing a scatterplot of mitochondrial gene expression, two histograms depicting read counts and feature counts (genes, transcripts, etc.), and finally a scatterplot of read-counts vs feature counts, which plots a smoothed regression line overlaid by the 95% confidence interval to provide the uncertainty about the regression line [19]. An additional tool is provided that uses *scater*’s (scater, RRID:SCR_015954) inbuilt ‘plotExprsFreqVsMean’ method, which plots gene expression frequency against mean expression level, in order to examine the effects of technical dropout

in the data. Finally, we provide methods to examine batch effect and other confounding factors in our QC-adhering data. A confounding factor might be any parameter introduced during library preparation or sequencing, such as the cells being from different sequencing runs, being handled by different technicians, being processed on different days, etc. Using *scater*'s inbuilt methods, the high-quality data are first normalised. Next, a Principle Component Analysis (PCA) is applied to the normalised data, which is then plotted. Points on the plot represent data that explain the maximal amount of variance, which can be annotated in relation to any column heading in the experiment annotation file. Categorical data may be given a unique shape or colour, whilst points for continuous data may be given scaled sizes. For example, points on the PCA plot may be coloured by sample, shaped by batch and sized by total features. As batch effects can be hidden in higher order components, we also provide a method to generate t-stochastic neighbour embedding (t-SNE) plots. Using normalised data, the t-SNE for the cells is first calculated and then plotted, again with the ability to shape, size, and colour points according to experimental parameters.

Filtering tool

We provide two alternative methods for filtering. In the first one the user manually selects cut-off parameters (usually informed by the plotting tools above), above or below which cells are removed if they do not reach the threshold. The metrics that can be filtered for are: the number of expressed genes, library size (calculated from the number of mapped reads), and percentage of reads mapped to mitochondrial genes. Cells failing these thresholds will be removed. The second method automatically removes cells that are categorised as outliers from PCA. This method works by identifying low-quality cells that have markedly different QC metrics from other cells. Both of the filtering methods are designed to be iterative and the

user has the option to re-run filtering from raw data, or refine filtering from a previous filtering step (Figure 1).

Future work

Although we do not include an exhaustive suite of tools, we aim to include ones that are being used widely in scRNA-seq QC. Future tools would most likely include improved outlier detection methods along with methods to account for cell-cycle heterogeneity.

Summary

A large number of tools are available to analyse scRNA-seq data, but many of them require quite advanced computational skills and resources to run. These requirements make it difficult to firstly learn the basics of scRNA-seq analyses and to then have the power to perform more complicated downstream analyses on typically large datasets (hundreds, or even thousands of samples). We have developed tools and training materials that make it easy to learn and run typical short-read scRNA-seq quality control steps and analyses. These tools can be used either on the command line in an intuitive, iterative manner, or can be integrated into the Galaxy platform, which will allow further downstream analyses with other tools.

Availability of supporting source code and requirements

- Project name: Wrappers for scater
- Project home page: https://github.com/galaxyproject/tools-iuc/tree/master/tools/scater
  Operating systems: Platform independent
- Programming language: R
- Other requirements: scater
- License: MIT

- RRID: SCR_017394

Availability of Supporting Data

All our code and wrappers, complete with installation instructions, tool help and an example workflow, are available under the MIT open-source license at https://github.com/galaxyproject/tools-iuc/tree/master/tools/scater and on the Galaxy ToolShed at https://toolshed.g2.bx.psu.edu/view/iuc/suite_scater/. Snapshots of the code are also available from the *GigaScience* GigaDB repository [20]. The tools can also be freely used at the UseGalaxy.eu public server (https://usegalaxy.eu). We have added a tutorial that covers a typical analysis workflow in Galaxy which can be found at https://training.galaxyproject.org/training-material/topics/transcriptomics/tutorials/scrna-scater-qc/tutorial.html

This resource has been submitted to SciCrunch.org under the RRID SCR_017394 and is tracked in bio.tools under software name ‘Galaxy scater’.

Abbreviations

HPC: High-Performance Computing; PCA: Principle Component Analysis; scRNA-seq: single-cell RNA sequencing; t-SNE: t-stochastic neighbour embedding

Competing interests

The authors declare that they have no competing interests.

Acknowledgements

The authors would like to acknowledge the EBI Gene Expression Group (EBI-GEG) for their helpful inspiration, comments and suggestions. The EBI-GEG together with ourselves and

other researchers are part of a larger collaborative effort to make Galaxy-based SC analysis more accessible to the community at https://singlecell.usegalaxy.eu/

Funding

This work was strategically funded by the BBSRC Core Strategic Programme Grants BBS/E/T/000PR9817, BBS/E/T/000PR9818, and BBS/E/T/000PR9819 and Core Capability Grant BBS/E/T/000PR9816 at the Earlham Institute.

References:

1. Tang, F., et al., *mRNA-Seq whole-transcriptome analysis of a single cell.* Nat Methods, 2009. **6**(5): p. 377-82 DOI: 10.1038/nmeth.1315.
2. Wagner, A., A. Regev, and N. Yosef, *Revealing the vectors of cellular identity with single-cell genomics.* Nat Biotechnol, 2016. **34**(11): p. 1145-1160 DOI: 10.1038/nbt.3711.
3. Bray, N.L., et al., *Near-optimal probabilistic RNA-seq quantification.* Nat Biotechnol, 2016. **34**(5): p. 525-7 DOI: 10.1038/nbt.3519.
4. Dobin, A., et al., *STAR: ultrafast universal RNA-seq aligner.* Bioinformatics, 2013. **29**(1): p. 15-21 DOI: 10.1093/bioinformatics/bts635.
5. Patro, R., et al., *Salmon provides fast and bias-aware quantification of transcript expression.* Nat Methods, 2017. **14**(4): p. 417-419 DOI: 10.1038/nmeth.4197.
6. Andrews, T.S. and M. Hemberg, *M3Drop: Dropout-based feature selection for scRNASeq.* Bioinformatics, 2018 DOI: 10.1093/bioinformatics/bty1044.
7. Butler, A., et al., *Integrating single-cell transcriptomic data across different conditions, technologies, and species.* Nat Biotechnol, 2018. **36**(5): p. 411-420 DOI: 10.1038/nbt.4096.

8. Ji, Z. and H. Ji, *TSCAN: Pseudo-time reconstruction and evaluation in single-cell RNA-seq analysis.* Nucleic Acids Res, 2016. **44**(13): p. e117 DOI: 10.1093/nar/gkw430.

9. Kiselev, V.Y., et al., *SC3: consensus clustering of single-cell RNA-seq data.* Nat Methods, 2017. **14**(5): p. 483-486 DOI: 10.1038/nmeth.4236.

10. Qiu, X., et al., *Reversed graph embedding resolves complex single-cell trajectories.* Nat Methods, 2017. **14**(10): p. 979-982 DOI: 10.1038/nmeth.4402.

11. Smith, T., A. Heger, and I. Sudbery, *UMI-tools: modeling sequencing errors in Unique Molecular Identifiers to improve quantification accuracy.* Genome Res, 2017. **27**(3): p. 491-499 DOI: 10.1101/gr.209601.116.

12. Wolf, F.A., P. Angerer, and F.J. Theis, *SCANPY: large-scale single-cell gene expression data analysis.* Genome Biol, 2018. **19**(1): p. 15 DOI: 10.1186/s13059-017-1382-0.

13. McCarthy, D.J., et al., *Scater: pre-processing, quality control, normalization and visualization of single-cell RNA-seq data in R.* Bioinformatics, 2017. **33**(8): p. 1179-1186 DOI: 10.1093/bioinformatics/btw777.

14. Gentleman, R.C., et al., *Bioconductor: open software development for computational biology and bioinformatics.* Genome Biol, 2004. **5**(10): p. R80 DOI: 10.1186/gb-2004-5-10-r80.

15. R Core Team, *R: A Language and Environment for Statistical Computing*. 2018. https://www.r-project.org/

16. Kwok, R., *Computing: Out of the hood.* Nature, 2013. **504**(7479): p. 319-21.

17. Afgan, E., et al., *The Galaxy platform for accessible, reproducible and collaborative biomedical analyses: 2018 update.* Nucleic Acids Res, 2018. **46**(W1): p. W537-W544 DOI: 10.1093/nar/gky379.

18. Batut, B., et al., *Community-Driven Data Analysis Training for Biology.* Cell Syst, 2018. **6**(6): p. 752-758 e1 DOI: 10.1016/j.cels.2018.05.012.

19. Wickham, H., *ggplot2: Elegant Graphics for Data Analysis*. 2016: Springer-Verlag New York.

20. Etherington GJ; Soranzo N; Mohammed S; Haerty W; Davey RP; Di Palma F (2019): Supporting data for "A Galaxy-based training resource for single-cell RNA-seq quality control and analyses" GigaScience Database. http://dx.doi.org/10.5524/100663

Figure 1

Click here to access/download;Figure;Figure1.jpg

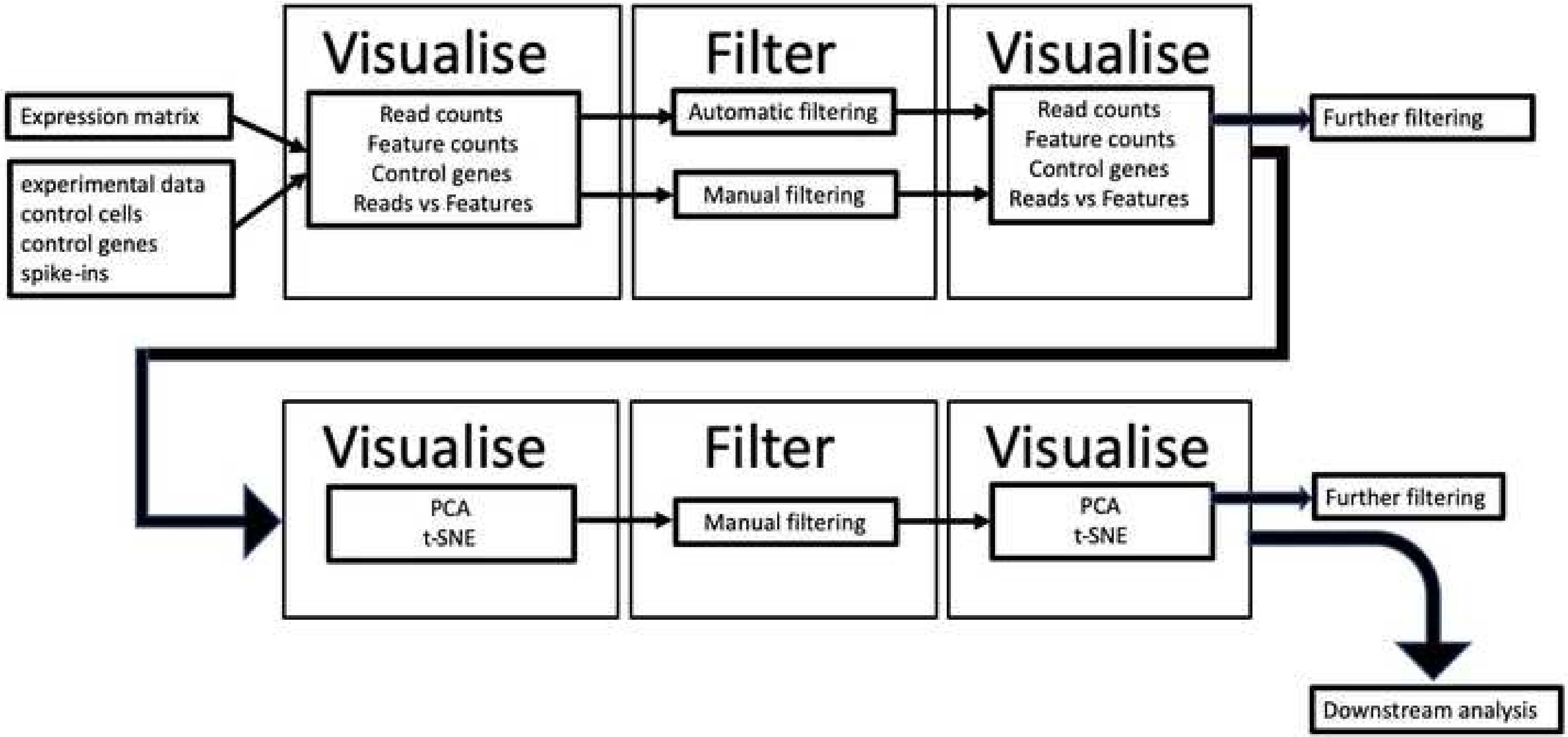

Figure 2

Click here to access/download Figure;Figure2.pdf

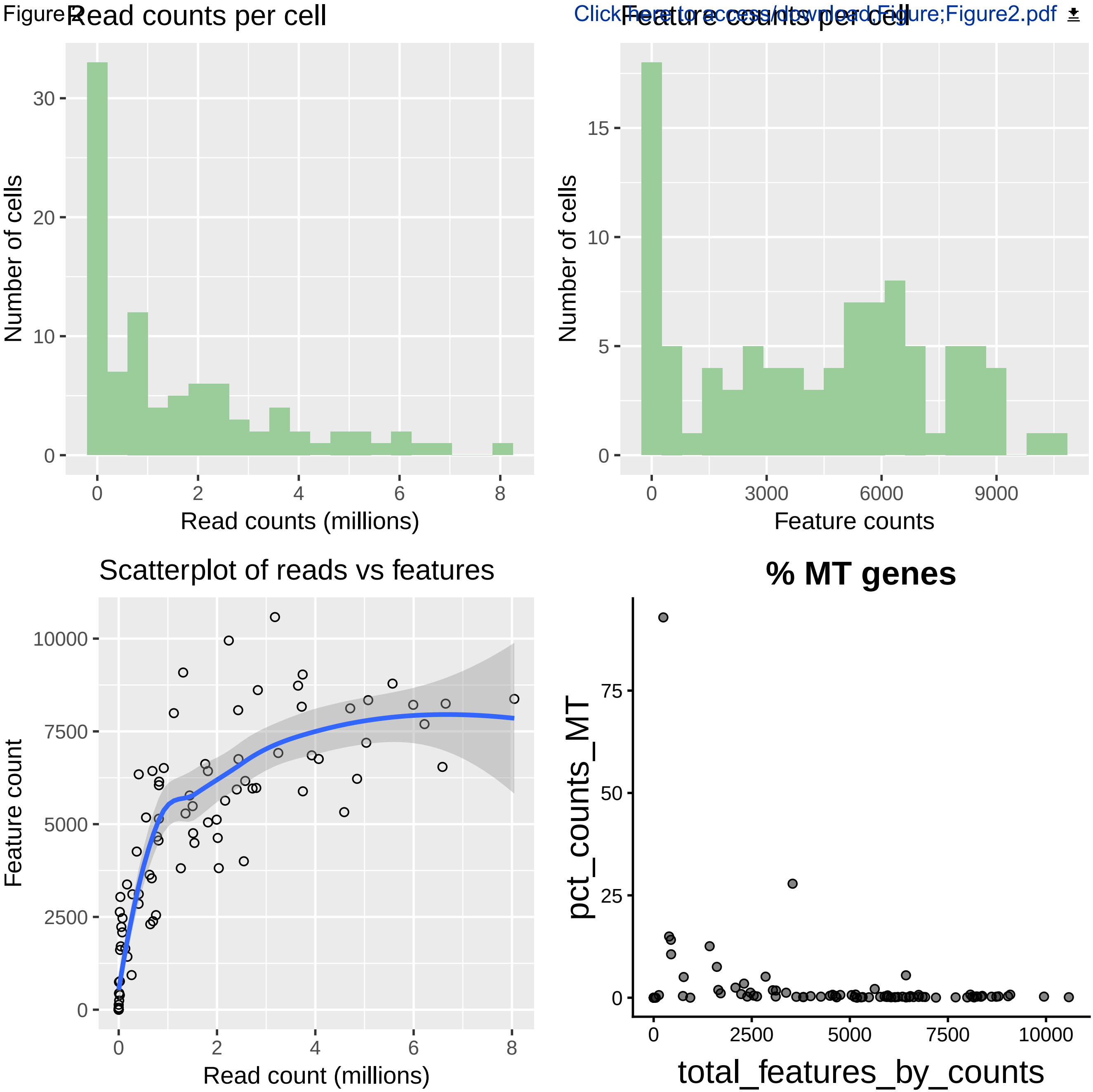

Figure 3

Click here to access/download;Figure;Figure3.pdf

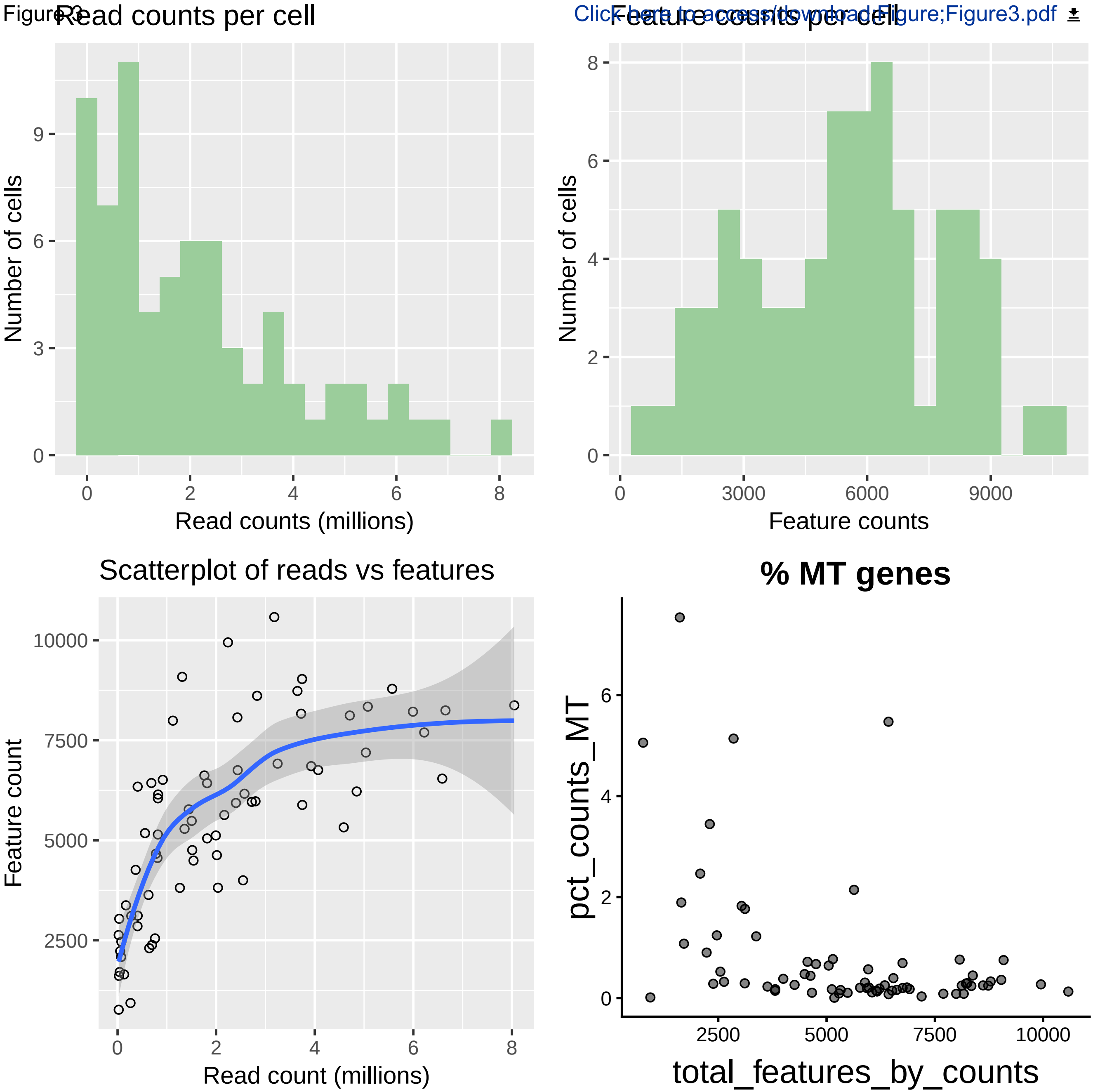

Click here to access/download
Supplementary Material
SupplementaryData.docx

Response to Reviewers
Click here to access/download;Personal Cover;ResponseToReviewers.docx

Dear GigaScience,
Thank you for the recent feedback from the three peer-reviewers. We'd like to thank them for taking the time to review and comment on the paper. The feedback was very complementary and constructive, and we hope you find the manuscript is now greatly improved.
As requested we have registered the software with SciCrunch and bio.tools and entered this information under 'Availability' in the manuscript.

We have addressed every point proposed by each of the reviewers as follows:

Reviewer #1.

The abstract gives the impression that the scripts performs a full analysis, which would include clustering, but the aim is actually to be a comprehensive quality control suite for the pre-analysis stage. The wording should reflect this more, especially in the abstract, as it could otherwise be misleading for researchers wishing to perform a full analysis. It is also not entirely clear which types of datasets this suite is targeting, since the field is steadily shifting towards 10X datasets which tend to have much higher sequencing depth and are less prone to noise and dropout events.

Response: We have added more emphasis on the quality-control aspect of the software. We have also specified 'short-read' sequencing data where appropriate to separate it from 10x/UMI data.

The abstract brings up the topic of "missing intuitive training materials" in the field of scRNA and the background section references the Galaxy Training Network (GTN) as a potential solution to this, but there does not yet appear to be training material for the suite in the GTN, nor an example workflow. The suite presented here would be a very welcome addition to the current scRNA tutorials as it would fill a vacancy between the pre-processing and downstream analysis tutorials. The code repository provides a README which is very comprehensive to what the suite can do, but this really would be better demonstrated in the GTN. The title of the paper should also reflect this, since though it does indeed provide tools that would be very beneficial for scRNA-seq training, it does not yet provide the training materials.

Response: We have added a tutorial and test data that covers a typical analysis workflow in Galaxy which will appear at https://training.galaxyproject.org/training-material/topics/transcriptomics/tutorials/scrna-scater-qc/tutorial.html after being reviewed and merged. In the meantime, it can be viewed at https://github.com/galaxyproject/training-material/pull/1559

- *More emphasis to the QC aspect of the tool should be given.*
- *Word changes:*

* "easy-to-use":
- Repetition of "easy-to-use" in the abstract.
- The same compound adjective appearing twice in succession feels slightly jarring to read.
* "assess, visualise, and quality control"
- Quality control is used as a verb here, please change.
- Perhaps: "perform quality control upon"
* "difficult to master the basics of scRNA-seq quality control and analysis"
- Given that this is more of a QC suite, the wording should be changed.
- Perhaps: "difficult to master the basics of scRNA-seq quality control and the later analysis."

Response: All these suggestions have been implemented.

2.2.2 Background

- The various different components of an scRNA-seq analysis were mentioned (pathway analysis, cell trajectories, type inference, pseudotime, dropouts, etc.) it would be useful to cite a paper that mentions these. I can highly recommend a 2016 paper from Wagner et al. ("Revealing the vectors of cellular identity with single-cell genomics" - <https://doi.org/10.1038/nbt.3711>) in this regard.

Response: Thanks for the useful reference. We've added this to the paper.

- The requirement of HPC resources to use these scripts does not feel warranted, since PCA is relatively trivial to compute on basic hardware. The main bottleneck for scRNA-seq analysis is the clustering step which this suite is upstream of.

Response: This is a very good point. Although we typically do these analyses on HPCs, they do not necessarily require such resources. We’ve moved this statement to earlier in the manuscript, where we talk about mapping reads to a reference and calculating the gene-expression matrix, processes which typically use lots of computational resources.

- Word changes:
* "due to dominance of the transcription profile of one population"
- (very minor change)
- Perhaps: "due to the transcription profile of one population dominating the"
* "door to identification of"
- Perhaps: "door to the identification of "

Response: All these suggestions have been implemented

** Reference [1]*
- Please place references at the end of the sentence.
** Reference [12]*
- Please place references at the end of the sentence.

Response: All references now appear at the end of the sentence in which they appear.

** "within the community"*
- Please state which community: bioinformaticians, cell biologists, etc.

Response: As we've moved the line about HPC's to just before this phrase, we've changed the whole sentence from "There are a number of tools available for this process (or variations of it) that are widely used within the community", to "There are a number of tools available that utilise HPCs to perform these processes (or variations of them) that are widely used by bioinformaticians"

** "Scater requires at least some experience of the Linux commandline or even more advanced experience of the R programming language"*

Response: We'd argue that 'commandline' is not a proper word. A search of GigaScience invariably has it spelled with a space 'command line' or hyphenated 'command-line' and never as a single word. For continuity, we've hyphenated it where it appears as a compound adjective (e.g "command-line tools"), or with a space when used otherwise (e.g. ""on the command line").

- (very minor)
- R packages can be run from RStudio and Jupyter notebooks, so Linux commandline is not a huge issue except when things go wrong. Perhaps change the wording to not make Linux appear as if it is the first issue that users may encounter.

Response: Good point. As stated, one could run scater without the command-line. We've removed the reference to command-line here and mentioned only R programming. Changed from "Like many of the other tools cited above, Scater requires at least some experience of the Linux command-line or even more advanced experience of the R programming language", to "Like many of the other tools, Scater requires at least some experience of the R programming language "

** "well over"*
- Please avoid using idioms.
** "or can be easily integrated with"*
- (very minor)
- Perhaps: "and can be easily integrated into"

Response: All suggestions have been implemented.

2.2.3 Results

- The "visualise-filter-visualise" paradigm is a really good one, and I would emphasise this more in other sections, especially the abstract. The use of the Loom format should also be mentioned in the abstract as it serves as a good inter-exchange format for other tools, extending the usability of this suite by making it more independent of the R ecosystem.

Response: We have added mentions of Loom and the 'visualise-filter-visualise' paradigm in the abstract.

- The step-by-step part is slightly too wordy, and much of the text here could be reduced by showing the various inputs and outputs of each step with a diagram. It would be extremely useful to include an image of the actual workflow or a flowchart making use of the "visualise-filter-visualise" paradigm, not only to accompany the step-by-step overview but perhaps also to demonstrate the potentially branching nature of such a workflow, where a user may run several different parameters in parallel and then collate and inspect their results, selecting the path that yielded the best results.

Response: We've added a new figure (now Figure 1) and cut down the text where possible in the step-by-step guide.

- Perhaps a slight de-emphasis of the commandline aspect of the suite would be in order, since the conda environments required to run them (r-optparse, bioconductor-loomexperiment, etc) are part of the macros.xml, and not in their own requirements.txt file for ease of commandline use.

Response: This should now be solved by the addition of the instructions for the creation of the conda environment on the command line.

- Since user convenience is a selling point of this suite, it would also be nice to see an image of the graphical (Galaxy) interface in action.

Response: We have added two supplementary figures of the Galaxy interface depicting before and after running the 'QC plot' tool.

- The environment required to run the tool should be given. The suite is reproducible in the sense that it lists a Scater version, but it does not provide the means to set up the environment. The macros.xml file lists a few more dependencies that may be required for the suite to function () and it would be good to provide these requirements in a text file that can be installed via conda.

Response: We have added a conda environment.yml file and provided instructions in the README.md on how to create and activate a conda environment from that file.

- It would be good to explain what the upper and lower bounds of the grey zone of the 'Scatterplot of reads vs features' are, and what they mean in context to the data.

Response: We have inserted an explanation of the regression line and 95% CI lines under the Plotting Tools section.

- Word changes:
** "further round"*
- Please avoid using idioms.
** "focussed"*
- Spelling: "focused"
** "calculate some metrics"*
- The 'some' part sounds vague. I would remove it.
- Perhaps just: "calculate metrics"
** "test the methods below"*
- Perhaps: "test the methods outlined below".

Response: All suggestions have been implemented.

2.2.4 Methods

- It would be useful to mention how the PCA separates the data and what parameters would constitute an outlier cell. Some tools use outlier cells to produce

Response: Some examples of what might cause outlier cells have been included along with a brief explanation of the plots.

- RaceID performs outlier detection, is it good to filter them out here? Why not later?
- Cell-cycle effect regression is noticeably absent. This is mentioned as a potential feature in a newer version of Scater in the vignette (<https://bioconductor.statistik.tu-dortmund.de/packages/3.3/bioc/vignettes/scater/inst/doc/vignette.html>) and it would be useful for the authors to mention this in a small future work paragraph.

Response: We have included a 'Future Work' section where we address features that we aim to improve/add methods.

It may also be useful to mention the future-proof aspects of Loom in such a paragraph.

Response: As we do not plan to work on Loom per se, we have expanded our introduction of Loom by adding "As Loom provides efficient access to arbitrary rows and columns, it scales well with the increasing size of high-throughput genomics data and is supported by nearly all programming languages."

- Word changes:
** "in our QC'd data"*
- Perhaps: "in our QC-adhering data"
** "the high-quality data is firstly normalised"*
- Perhaps: "the high-quality data is first normalised"
** "2 alternative methods"*
- Perhaps: "two alternative methods", unless the absolute number of methods is significant?
** "refine filtering from a previous filtering step"*

Response: All suggestions have been implemented.

** An image of this would be greatly appreciated. See comments in Results.*

Response: See new Figure 1.

2.2.5 Summary

- Large datasets are mentioned, but not defined.

Response: We have inserted a definition of “hundreds, or even thousands of samples”

Does this suite scale well with 10X datasets, or is it aimed more at the smaller noisier sets with low sequencing depth?

Response: We have inserted references to 'short-read sequencing' to differentiate from 10x Genomics. We have (as yet) to apply it to 10x data.

- Word changes:
** "and then have the power"*
- Perhaps: "and to then have the power"
** "a typical workflow"*
- Perhaps: "an example workflow"

Response: All suggestions have been implemented.

Reviewer #2:

Figure 2 is supposed to be a nicely filtered version of Figure 1, however it doesn't look that much better.
The 'filtration' didn't remove a load of cells with high mt content or low read count.

Response: We respectfully disagree. In the examples, we removed low quality cells that contain less than 10,000 mapped reads or have more than 8% of their expressed features from mitochondrial genes. In the first plot (Read counts per cell), the number of cells with low read counts (<400,000) has fallen from more than 30 to 10. Similarly, this lowers the number of cells with a low number of expressed genes (<1000) from 18 to 1. Overall, using these parameters we have 71 high-quality cells remaining from the initial 95. To clarify the changes in the plots, we have added the following two sentences to the legend of Figure 2 (now Figure 3 after input from another reviewer). "The data shown in Figure 2 has been filtered by removing cells that have less than 10,000 mapped reads and cells that have more than 8% of their expressed features from mitochondrial DNA."... "From the original 95 cells in the pre-filtered dataset, we have 71 high-quality cells remaining."

Also 'feature' would be more understandable as 'UMI' and images showing what they are talking about (i.e. PCA/batch effect) would be helpful.

Response: In the plots, 'Features' are any type of feature listed in column 3 of a GFF/GTF file https://www.ensembl.org/info/website/upload/gff.html As a user may be looking at expressed genes or expressed transcripts, (or mRNA, TSSs, exons, for that matter) we decided to leave the x-axis of the 'Feature counts per cell' as 'features counts' as 'feature' is the technically correct term. UMI counts (used as alternative to read counts) are used to calculate the expression of features and so would not be a suitable description of this axis. Also, the expression data used in the paper is from read-counts, not UMI counts.

Finally, I think pre/post filtration should be in the same image so it's easier to compare.

Response: The plots are the output from the software, which demonstrate the core 'visualise-filter-visualise' paradigm of the paper. We feel it would be misleading to place them in the same image as this is not how they would appear when the software was used. The figures are placed sequentially in the paper, so we don't feel comparing them will be problematic.

<u>Reviewer #3:</u>

Overview

It would have been nice to include a UMAP or t-SNE plot as part of the package. The PCA plots are sometimes useful for finding large batch effects, but often times those batch effects are hidden in higher order components and you'd miss them by just looking at components 1 and 2 in the PCA but you'd see the effects with a UMAP or t-SNE plot. An alternative would be to do a principal components correlation; you correlate metadata and derived measures such as number of genes detected, total counts, etc with the principal components and generate a heatmap of correlation with the components. This lets you look at many more components and find batch effects or other non-biologically interesting effects in the higher order components. I appreciate the intent to focus only on the first few steps of the analysis, but being able to look at the more higher-order effects is an essential part of quality controlling single-cell RNA-seq data so I wish the option was included.

Response: Thank you for the feedback. We have implemented t-SNE as a new plotting tool and added a few sentences explaining how it works: "As batch effects can be hidden in higher order components, we also provide a method to generate t-stochastic neighbour embedding (t-SNE) plots. Using normalised data, the t-SNE for the cells is first calculated and then plotted, again with the ability to shape, size, and colour points according to experimental parameters."

Below are some specific issues with the text:

# Galaxy tool issues
## Warning with plot expression frequency tour
Warning: The file 'Type' was set to 'loom' but the file does not appear to be of that type
WARNING:galaxy.datatypes.binary:<galaxy.datatypes.binary.Loom object at 0x2b163ace8590>, set_meta Exception: "Unable to open object (object 'layers' doesn't exist)

Response: This warning appears when uploading a Loom file to Galaxy, created by the Bioconductor LoomExperiment package. It is harmless, but we have nonetheless opened a pull request to the Galaxy GitHub repository to fix the issue: https://github.com/galaxyproject/galaxy/pull/8560

Clarity/style suggestions

## General comments
1. I'm pretty sure scater is not capitalized-- they don't capitalize it in the package name, nor the vignettes, so I think the convention is to leave it as lowercase.

Response: We have ensured that scater is spelled in lower-case and italicised throughout.

2. I think it is often very useful to have the option to plot the genes detected vs expression level and other plots like that in log scale when you are going to pick cutoffs. For the small

number of cells in the example, it doesn't matter, but it matters a lot when you have a large number of cells.

Response: We have added this as an optional feature in the R code and created a boolean choice in the Galaxy wrapper.

3. https://github.com/galaxyproject/tools-iuc/tree/master/tools/scater is stated as being MIT licensed, but a LICENSE file does not exist there.

Response: A LICENSE file is not needed in the `scater` subdirectory because the one in the main directory applies by default: https://github.com/galaxyproject/tools-iuc/blob/master/LICENSE

4. scater itself is GPLed so I think you could be stuck with the GPL license if you are using its code, I'm not sure how that works.

Response: We own the copyright and apply the MIT license only on the Galaxy wrapper code (i.e. XML and Python files in the https://github.com/galaxyproject/tools-iuc/tree/master/tools/scater). Given that the wrappers use scater through its command-line interface, the two can be considered separate "programs", as explained in https://www.gnu.org/licenses/gpl-faq.html#MereAggregation

## Sentence replacements
1. Typically, reads generated from cells in scRNA-seq experiments are mapped to a reference genome and then an expression matrix, calculated from the number of reads that are allocated to each gene or transcript, is produced -> Typically, reads generated from cells in scRNA-seq experiments are mapped to a reference genome and then an expression matrix is, calculated from the number of reads that are allocated to each gene or transcript.
2. scRNA-seq data is inherently 'noisy' -> scRNA-seq data is inherently noisy.
3. Here raw data, before being filtered is plotted. -> Raw data, before filtering, is being plotted in this figure.
4. Summary. -> Summary (The heading has a period after it, unlike the rest of the headings).
5. Filtering tool. -> Filtering tool (Similar issue to the above)
6. We provide a filtering tool with 2 alternative methods. -> We provide two alternate methods for filtering.
7. A large number of tools are available to analyse scRNA-seq data. Many of them require quite advanced computational skills and resources to run. -> A large number of tools are available to analyse scRNA-seq data but many of them require quite advanced computational skills and resources to run.
8. The Competing interests heading is indented and should not be.

Response: All suggestions have been implemented.
