# Supplementary material for: A Galaxy-based training resource for single-cell RNA-sequencing quality control and analyses
Source: Gigascience. 2019 Dec 11;8(12):giz144. doi: 10.1093/gigascience/giz144 (PMC6905351; doi:10.1093/gigascience/giz144)
Supplement: giz144_Supplemental_Figures [file giz144_supplemental_figures.docx]

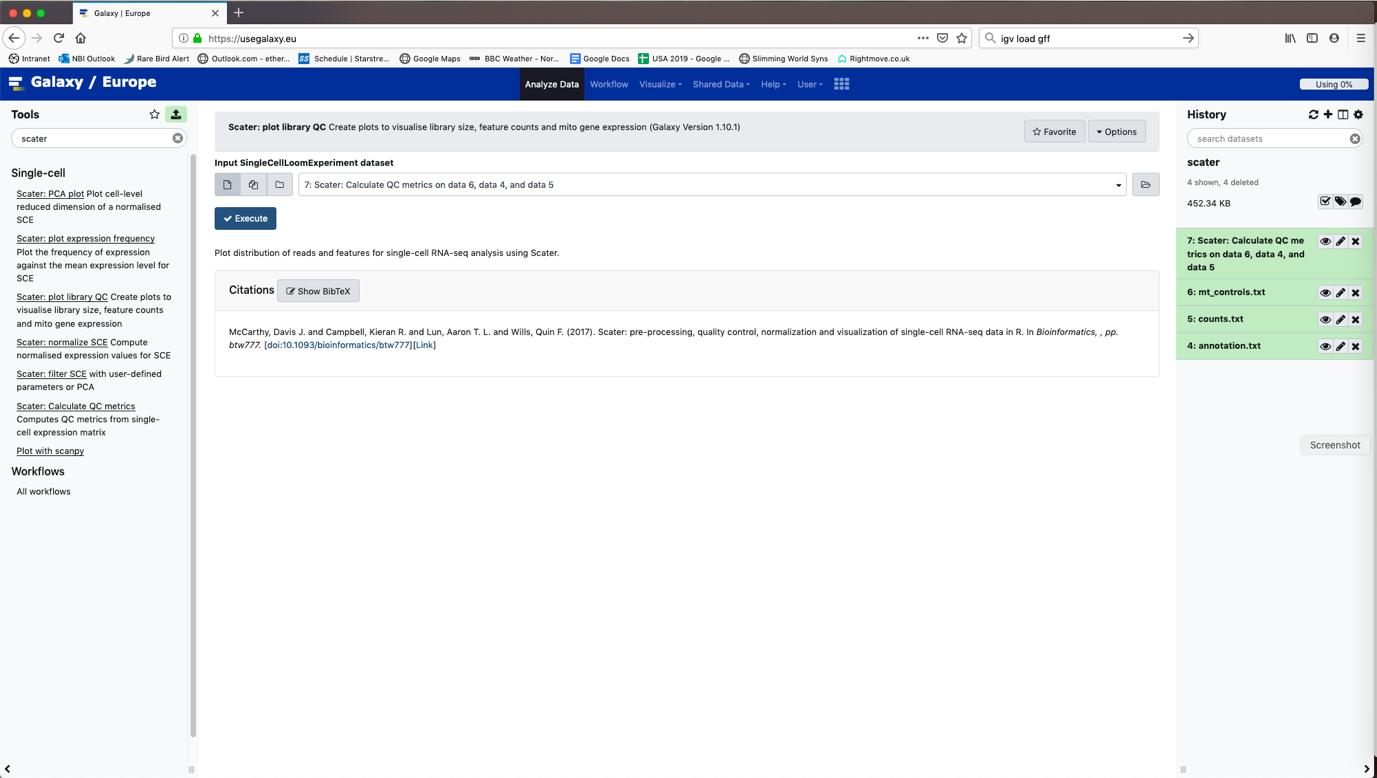


Supplementary Figure S1. The Galaxy interface for the scater tools, shown in the left-hand column. Data is shown in green in the history panel to the right of the interface. Here, an expression matrix, cell annotation and mitochondrial control genes have been used to create QC-ready data by using the ‘Calculate QC metrics’ tool. The ‘plot library QC’ tool has now been selected and the user merely selects the input file to run the tool


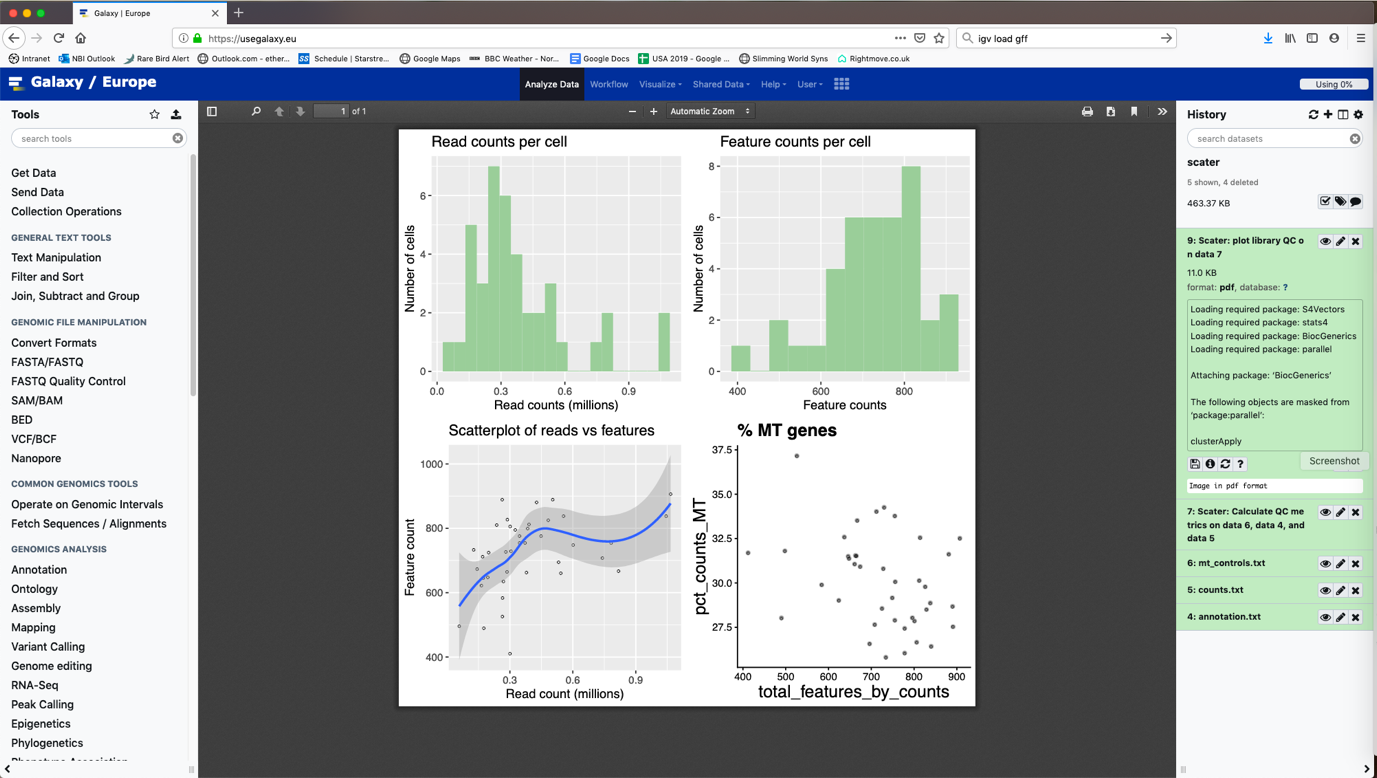


Supplementary Figure S2. The output of the ‘plot library QC’ tool can be displayed within Galaxy. The plot is used to inform parameters for the subsequent filtering steps.
