# Supplementary material for: A Galaxy-based training resource for single-cell RNA-sequencing quality control and analyses
Source: Gigascience. 2019 Dec 11;8(12):giz144. doi: 10.1093/gigascience/giz144 (PMC6905351; doi:10.1093/gigascience/giz144)
Supplement: giz144_Response_to_Reviewer_Comments_Original_Submission [file giz144_response_to_reviewer_comments_original_submission.pdf]

Author's Response To Reviewer Comments

Close

Please see personal cover file, 'ResponseToReviewers'.

Close
