# Supplementary material for: A Galaxy-based training resource for single-cell RNA-sequencing quality control and analyses
Source: Gigascience. 2019 Dec 11;8(12):giz144. doi: 10.1093/gigascience/giz144 (PMC6905351; doi:10.1093/gigascience/giz144)
Supplement: giz144_Reviewer_1_Report_Original_Submission — Mehmet Tekman -- 8/6/2019 Reviewed [file giz144_reviewer_1_report_original_submission.pdf]

Reviewer Report

Title: A Galaxy-based training resource for single-cell RNA-seq quality control and analyses

Version: Original Submission **Date:** 8/6/2019

Reviewer name: Mehmet Tekman

Reviewer Comments to Author:

Review: A Galaxy-based training resource for single-cell RNA-seq quality control and analyses

---

Table of Contents

---

1. General Comments
2. Specific Comments for Revision
.. 1. Major
..... 1. Scope of the Analysis
..... 2. Training Materials
.. 2. Minor
..... 1. Abstract
..... 2. Background
..... 3. Results
..... 4. Methods
..... 5. Summary

1 General Comments
====================

This is a well written article which deals with the crucial quality control (QC) stage of scRNA-seq analysis, providing a good introduction to the many tribulations a researcher may encounter when first starting this type of analysis. It provides a set of script-like modules based on the Scater package which performs QC in an iterative manner using a "visualise-filter-visualise" inspection paradigm that succinctly captures the QC process adopted by many in the field.
The main feature of the modules is to remove low-quality cell types which would add unwanted noise to the later clustering, and to remove technical and biological variation. The interchange format between modules is the Loom format which facilitates the exchange between different single-cell tools and moves away from the more R-based formats that dominated the package ecosystem just one year prior.
The modules are also wrapped into the Galaxy framework to facilitate

the use of the modules for less commandline-oriented researchers, and for them to also make use of the excellent teaching and training resources that they can make use of to further their analysis.
The quality of the scripts, both in terms of code content and the suitability of the analysis are up to a good standard, and provided that a Galaxy workflow is created and that a tutorial for the training material is written, the suite described here would be a very welcome addition to the Galaxy Training Network.

2 Specific Comments for Revision
==

2.1 Major

2.1.1 Scope of the Analysis

The abstract gives the impression that the scripts performs a full analysis, which would include clustering, but the aim is actually to be a comprehensive quality control suite for the pre-analysis stage. The wording should reflect this more, especially in the abstract, as it could otherwise be misleading for researchers wishing to perform a full analysis. It is also not entirely clear which types of datasets this suite is targeting, since the field is steadily shifting towards 10X datasets which tend to have much higher sequencing depth and are less prone to noise and dropout events.

2.1.2 Training Materials

The abstract brings up the topic of "missing intuitive training materials" in the field of scRNA and the background section references the Galaxy Training Network (GTN) as a potential solution to this, but there does not yet appear to be training material for the suite in the GTN, nor an example workflow. The suite presented here would be a very welcome addition to the current scRNA tutorials as it would fill a vacancy between the pre-processing and downstream analysis tutorials. The code repository provides a README which is very comprehensive to what the suite can do, but this really would be better demonstrated in the GTN. The title of the paper should also reflect this, since though it does indeed provide tools that would be very beneficial for scRNA-seq training, it does not yet provide the training materials.

2.2 Minor

2.2.1 Abstract

- More emphasis to the QC aspect of the tool should be given.
- Word changes:
  * "easy-to-use":
   - Repetition of "easy-to-use" in the abstract.
   - The same compound adjective appearing twice in succession feels slightly jarring to read.
  * "assess, visualise, and quality control"
   - Quality control is used as a verb here, please change.
   - Perhaps: "perform quality control upon"
  * "difficult to master the basics of scRNA-seq quality control and analysis"
   - Given that this is more of a QC suite, the wording should be changed.
   - Perhaps: "difficult to master the basics of scRNA-seq quality control and the later analysis."

2.2.2 Background

- I thoroughly enjoyed reading this section as it served as an excellent introduction to the field of single-cell genomics, clearly aimed for a specific audience in mind. It is written with a clarity of purpose and provides historical context to the field that is free from the technical jargon that plagues many of the other publications that are primarily aimed at those who are already in the field. I especially enjoyed the paragraph that mentions the "plethora of online help forums" aimed more at programmers, whilst resources for biologists being relatively few.
- The various different components of an scRNA-seq analysis were mentioned (pathway analysis, cell trajectories, type inference, pseudotime, dropouts, etc.) it would be useful to cite a paper that mentions these. I can highly recommend a 2016 paper from Wagner et al. ("Revealing the vectors of cellular identity with single-cell genomics" -) in this regard.
- The requirement of HPC resources to use these scripts does not feel warranted, since PCA is relatively trivial to compute on basic hardware. The main bottleneck for scRNA-seq analysis is the clustering step which this suite is upstream of.
- Word changes:
  * "due to dominance of the transcription profile of one population"
   - (very minor change)
   - Perhaps: "due to the transcription profile of one population dominating the"
  * Reference [1]

- Please place references at the end of the sentence.
* "door to identification of"
 - Perhaps: "door to the identification of "
* "within the community"
 - Please state which community: bioinformaticians, cell biologists, etc.
* Reference [12]
 - Please place references at the end of the sentence.
* "Scater requires at least some experience of the Linux commandline or even more advanced experience of the R programming language"
 - (very minor)
 - R packages can be run from RStudio and Jupyter notebooks, so Linux commandline is not a huge issue except when things go wrong. Perhaps change the wording to not make Linux appear as if it is the first issue that users may encounter.
* "well over"
 - Please avoid using idioms.
* "or can be easily integrated with"
 - (very minor)
 - Perhaps: "and can be easily integrated into"

2.2.3 Results

- The "visualise-filter-visualise" paradigm is a really good one, and I would emphasise this more in other sections, especially the abstract. The use of the Loom format should also be mentioned in the abstract as it serves as a good inter-exchange format for other tools, extending the usability of this suite by making it more independent of the R ecosystem.
- The step-by-step part is slightly too wordy, and much of the text here could be reduced by showing the various inputs and outputs of each step with a diagram. It would be extremely useful to include an image of the actual workflow or a flowchart making use of the "visualise-filter-visualise" paradigm, not only to accompany the step-by-step overview but perhaps also to demonstrate the potentially branching nature of such a workflow, where a user may run several different parameters in parallel and then collate and inspect their results, selecting the path that yielded the best results.
- Perhaps a slight de-emphasis of the commandline aspect of the suite would be in order, since the conda environments required to run them (r-optparse, bioconductor-loomexperiment, etc) are part of the macros.xml, and not in their own requirements.txt file for ease of

commandline use.
- Since user convenience is a selling point of this suite, it would
  also be nice to see an image of the graphical (Galaxy) interface in
  action.
- The environment required to run the tool should be given. The suite
  is reproducible in the sense that it lists a Scater version, but it
  does not provide the means to set up the environment. The macros.xml
  file lists a few more dependencies that may be required for the
  suite to function () and it would be good to provide these
  requirements in a text file that can be installed via conda.
- It would be good to explain what the upper and lower bounds of the
  grey zone of the 'Scatterplot of reads vs features' are, and what
  they mean in context to the data.
- Word changes:
  * "further round"
   - Please avoid using idioms.
  * "focussed"
   - Spelling: "focused"
  * "calculate some metrics"
   - The 'some' part sounds vague. I would remove it.
   - Perhaps just: "calculate metrics"
  * "test the methods below"
   - Perhaps: "test the methods outlined below".

2.2.4 Methods

- It would be useful to mention how the PCA separates the data and
  what parameters would constitute an outlier cell. Some tools use
  outlier cells to produce
  - RaceID performs outlier detection, is it good to filter them out
    here? Why not later?
- Cell-cycle effect regression is noticeably absent. This is mentioned
  as a potential feature in a newer version of Scater in the vignette
  ( )
  and it would be useful for the authors to mention this in a small
  future work paragraph. It may also be useful to mention the
  future-proof aspects of Loom in such a paragraph.
- Word changes:
  * "in our QC'd data"
   - Perhaps: "in our QC-adhering data"
  * "the high-quality data is firstly normalised"
   - Perhaps: "the high-quality data is first normalised"
  * "2 alternative methods"

- Perhaps: "two alternative methods", unless the absolute number of methods is significant?
* "refine filtering from a previous filtering step"
  * An image of this would be greatly appreciated. See comments in Results.

2.2.5 Summary

- Large datasets are mentioned, but not defined. Does this suite scale well with 10X datasets, or is it aimed more at the smaller noisier sets with low sequencing depth?
- Word changes:
  * "and then have the power"
    - Perhaps: "and to then have the power"
  * "a typical workflow"
    - Perhaps: "an example workflow"

Level of Interest

Please indicate how interesting you found the manuscript: Choose an item.

Quality of Written English

Please indicate the quality of language in the manuscript: Choose an item.

Declaration of Competing Interests

Please complete a declaration of competing interests, considering the following questions:

- Have you in the past five years received reimbursements, fees, funding, or salary from an organisation that may in any way gain or lose financially from the publication of this manuscript, either now or in the future?
- Do you hold any stocks or shares in an organisation that may in any way gain or lose financially from the publication of this manuscript, either now or in the future?
- Do you hold or are you currently applying for any patents relating to the content of the manuscript?
- Have you received reimbursements, fees, funding, or salary from an organization that holds or has applied for patents relating to the content of the manuscript?
- Do you have any other financial competing interests?
- Do you have any non-financial competing interests in relation to this paper?

If you can answer no to all of the above, write 'I declare that I have no competing interests' below. If your reply is yes to any, please give details below.

I declare that I have no competing interests

I agree to the open peer review policy of the journal. I understand that my name will be included on my report to the authors and, if the manuscript is accepted for publication, my named report including any attachments I upload will be posted on the website along with the authors' responses. I agree for my report to be made available under an Open Access Creative Commons CC-BY license (http://creativecommons.org/licenses/by/4.0/). I understand that any comments which I do not wish to be included in my named report can be included as confidential comments to the editors, which will not be published.

Choose an item.

To further support our reviewers, we have joined with Publons, where you can gain additional credit to further highlight your hard work (see: https://publons.com/journal/530/gigascience). On publication of this paper, your review will be automatically added to Publons, you can then choose whether or not to claim your Publons credit. I understand this statement.

Yes Choose an item.
