# Supplementary material for: A Galaxy-based training resource for single-cell RNA-sequencing quality control and analyses
Source: Gigascience. 2019 Dec 11;8(12):giz144. doi: 10.1093/gigascience/giz144 (PMC6905351; doi:10.1093/gigascience/giz144)
Supplement: giz144_Reviewer_3_Report_Original_Submission — Rory Kirchner -- 8/16/2019 Reviewed [file giz144_reviewer_3_report_original_submission.pdf]

Reviewer Report

Title: A Galaxy-based training resource for single-cell RNA-seq quality control and analyses

Version: Original Submission **Date:** 8/16/2019

Reviewer name: Rory Kirchner

Reviewer Comments to Author:

Overview
This work isn't groundbreaking, but good work doesn't need to be groundbreaking, it needs to be useful, and I think this is useful. There is a demand to make it easier for informatics-naive researchers to analyze all types of NGS data and single-cell RNA-seq is in particularly high demand. This work correctly points out that iterating visualize-filter-visualize loop is important when looking at single data. In addition the filtering step often requires expert biological knowledge of the particular experiment, something that is impossible for a dedicated informatician to know for all experiments they are analyzing, so having the wet-lab researcher be able to do this part themselves saves a lot of back and forth. I've analyzed dozens of single-cell RNA sequencing experiments and this step is often one of the most needlessly time consuming.

Integrating scater into the Galaxy toolshed is good, practical choice. Many informaticians, myself included, got started by using Galaxy, it is a great way for a computationally-naive researcher to try out tools. scater is a flexible way to do the initial analysis of single-cell RNA-seq data and once users are familiar with it, they can easily transition off of Galaxy once they are familiar with how to use scater. scater is also a well maintained, actively developed tool.

I tested all of the functions you integrated and they worked well, barring a few warnings. Including a toy dataset to test was helpful in reviewing the paper, thank you for doing that.

It would have been nice to include a UMAP or t-SNE plot as part of the package. The PCA plots are sometimes useful for finding large batch effects, but often times those batch effects are hidden in higher order components and you'd miss them by just looking at components 1 and 2 in the PCA but you'd see the effects with a UMAP or t-SNE plot. An alternative would be to do a principal components correlation; you correlate metadata and derived measures such as number of genes detected, total counts, etc with the principal components and generate a heatmap of correlation with the components. This lets you look at many more components and find batch effects or other non-biologically interesting effects in the higher order components. I appreciate the intent to focus only on the first few steps of the analysis, but being able to look at the more higher-order effects is an essential part of quality controlling single-cell RNA-seq data so I wish the option was included.

Below are some specific issues with the text:

Galaxy tool issues

Warning with plot expression frequency tour

Warning: The file 'Type' was set to 'loom' but the file does not appear to be of that type
WARNING:galaxy.datatypes.binary:, set_meta Exception: "Unable to open object (object 'layers' doesn't exist)

Clarity/style suggestions
General comments
1. I'm pretty sure scater is not capitalized-- they don't capitalize it in the package name, nor the vignettes, so I think the convention is to leave it as lowercase.
2. I think it is often very useful to have the option to plot the genes detected vs expression level and other plots like that in log scale when you are going to pick cutoffs. For the small number of cells in the example, it doesn't matter, but it matters a lot when you have a large number of cells.
3. https://github.com/galaxyproject/tools-iuc/tree/master/tools/scater is stated as being MIT licensed, but a LICENSE file does not exist there.
4. scater itself is GPLed so I think you could be stuck with the GPL license if you are using its code, I'm not sure how that works.
Sentence replacements
1. Typically, reads generated from cells in scRNA-seq experiments are mapped to a reference genome and then an expression matrix, calculated from the number of reads that are allocated to each gene or transcript, is produced -> Typically, reads generated from cells in scRNA-seq experiments are mapped to a reference genome and then an expression matrix is, calculated from the number of reads that are allocated to each gene or transcript.
2. scRNA-seq data is inherently 'noisy' -> scRNA-seq data is inherently noisy.
3. Here raw data, before being filtered is plotted. -> Raw data, before filtering, is being plotted in this figure.
4. Summary. -> Summary (The heading has a period after it, unlike the rest of the headings).
5. Filtering tool. -> Filtering tool (Similar issue to the above)
6. We provide a filtering tool with 2 alternative methods. -> We provide two alternate methods for filtering.
7. A large number of tools are available to analyse scRNA-seq data. Many of them require quite advanced computational skills and resources to run. -> A large number of tools are available to analyse scRNA-seq data but many of them require quite advanced computational skills and resources to run.
8. The Competing interests heading is indented and should not be.
Thank you! Integrating scater into Galaxy is useful to the bioinformatics community, and I'm glad you wrote it up.

Level of Interest

Please indicate how interesting you found the manuscript: Choose an item.

Quality of Written English

Please indicate the quality of language in the manuscript: Choose an item.

Declaration of Competing Interests

Please complete a declaration of competing interests, considering the following questions:

- Have you in the past five years received reimbursements, fees, funding, or salary from an organisation that may in any way gain or lose financially from the publication of this manuscript, either now or in the future?
- Do you hold any stocks or shares in an organisation that may in any way gain or lose financially from the publication of this manuscript, either now or in the future?
- Do you hold or are you currently applying for any patents relating to the content of the manuscript?
- Have you received reimbursements, fees, funding, or salary from an organization that holds or has applied for patents relating to the content of the manuscript?
- Do you have any other financial competing interests?
- Do you have any non-financial competing interests in relation to this paper?

If you can answer no to all of the above, write 'I declare that I have no competing interests' below. If your reply is yes to any, please give details below.

I declare that I have no competing interests.

I agree to the open peer review policy of the journal. I understand that my name will be included on my report to the authors and, if the manuscript is accepted for publication, my named report including any attachments I upload will be posted on the website along with the authors' responses. I agree for my report to be made available under an Open Access Creative Commons CC-BY license (http://creativecommons.org/licenses/by/4.0/). I understand that any comments which I do not wish to be included in my named report can be included as confidential comments to the editors, which will not be published.

Choose an item.

To further support our reviewers, we have joined with Publons, where you can gain additional credit to further highlight your hard work (see: https://publons.com/journal/530/gigascience). On publication of this paper, your review will be automatically added to Publons, you can then choose whether or not to claim your Publons credit. I understand this statement.

Yes Choose an item.
